# Heart function and thoracic aorta gene expression profiling studies of ginseng combined with different herbal medicines in eNOS knockout mice

**DOI:** 10.1038/s41598-017-15819-2

**Published:** 2017-11-13

**Authors:** Yuchen Qian, Pan Li, Bin Lv, Xiaoqing Jiang, Ting Wang, Han Zhang, Xiaoying Wang, Xiumei Gao

**Affiliations:** 1 State Key Laboratory of Modern Chinese Medicine, Tianjin University of Traditional Chinese Medicine, Tianjin, 300193 China; 2 College of Traditional Chinese Medicine, Tianjin University of Traditional Chinese Medicine, Tianjin, 300193 China

## Abstract

Ginseng, a popular herbal remedy, is often used in combination with other drugs to achieve the maximum therapeutic response. Shenfu (SFI) and Shenmai injection (SMI) have been widely used to treat cardiovascular disease in China. Our study explored the cardiovascular protection of SFI and SMI in eNOS knockout mice to investigate the differences and similarities of the two ginseng-combinations. Transthoracic echocardiography was performed to evaluate the left ventricular structure and function at baseline and 3, 7, and 14 days after drug administration. Agilent Gene Expression microarrays were used to demonstrate the gene expression profiling of the thoracic aorta. Ingenuity Pathway Analysis was performed to evaluate the mechanism improved by SFI and SMI in eNOS knockout mice. Both SFI and SMI could modulate Gadd45 Signaling from TOP15 canonical pathways. Moreover, SFI showed a better effect in the early treatment stage and improved myocardial function via GATA4, GATA6 and COL3A1. Meanwhile, SMI exerted better protective effects at the chronic stage, which may be related to endothelium protection by VEGFA and ACE. The advantage of multi-target by drug combination in progression of complex diseases should be noticed. The appropriate adjustment of drug combination could lead to a better accurate medical care in clinic.

## Introduction

Ginseng (Panax ginseng C.A. Meyer), which belongs to the genus Panax of the family *Araliaceae*, was first recorded in the oldest Chinese medical material *Shen Nong Ben Cao Jing* dating back to the 2nd century AD^1^. Ginseng has been used for health-related purposes for at least 2,000 years and has been among the top 10 selling herbal supplements in the United States over the past decade^2^. Ginseng is used to improve general well-being and relieve various health problems, such as cardiovascular disorders, respiratory disorders, and depression. Today, ginseng is widely used in Asia to treat cardiovascular diseases (CVD)^3^. Studies have shown that ginseng can inhibit cardiomyocyte hypertrophy and heart failure (HF)^4^ and prevent cardiac dysfunction^5^. Experimental studies have also revealed that ginseng can improve ischemic and reperfusion injury to the heart in a variety of animal models^6^. However, many complex physiological processes, such as inflammation^7^, oxidative stress^8^, and apoptosis are involved in CVD^9^. Medical science has long realized that the pathogenesis and progression of diseases are too complex for single drug treatment^10^. Therefore, herbal combination should be used to enhance curative effects, reduce toxicity, expand therapeutic range, adapt to complex disease, and prevent drug poisoning. Through a flexible combination, it can adjust the dynamic balance of the body in many ways, moreover, the advantages of adapting to the diversity of pathological changes are very prominent^11,12^. In China, ginseng is often used in combination with other herbal medicines to achieve maximum therapeutic response. In Chinese Pharmacopoeia (2015 edition), two ginseng-based injections are used to treat CVD; one is Shenfu Injection (SFI), which is ginseng with Radix Aconiti lateralis Preparata, and the other is Shenmai Injection (SMI), ginseng with Ophiopogon japonicus^13,14^.

SFI originates from Shenfu decoction, a well-known traditional herbal prescription recorded in *Sheng Ji Zong Lu* the 1100 s. SFI injection was officially approved in 1987 by the China Food and Drug Administration (CFDA)^15^. SFI is used to treat shock, congestive heart-failure (CHF) and arrhythmia and has been widely used clinically with nearly 600 million RMB in annual sales. SFI mainly contains ginsenosides and aconites^16^. Pharmacology research indicates that SFI can strengthen the heart, improve heart function^13^, and protect against post-resuscitation lung injury^17^. SMI is derived from a traditional decoction named Shengmai San that was prescribed in 1100s^15^. Identification of ingredients from SMI is in Supplementary File 1. MI was approved by the CFDA in 1995 and is used for the treatment of coronary heart disease^18^, chronic pulmonary heart disease^19^ and viral myocarditis^20^.

Our previous meta-analysis found that both SFI and SMI are effective in treating coronary heart disease and HF^21^. According to the system review, it is more inclined to use SFI in the treatment of acute HF in clinic. One the other hand, SMI is better in chronic stable angina patents. However, the complicated CVD leads to a promiscuous clinic application the two medicines. To date, there has been no strict standard distinction between SFI and SMI in clinical applications. No experimental research compares the different mechanisms of action between ginseng combined with Radix Aconiti lateralis Preparata and ginseng combined with Ophiopogon japonicus to understand the different actions of SFI and SMI in clinic.

By the reason of SFI showed significantly better effect than SMI in increasing myocardium function by the reason of ginseng combined with Aconiti lateralis, we major focused on the other side of CVD, endothelium. Keeping the artery endothelium and its nitric oxide (NO) synthesis intactness is crucial to maintain a normal vascular function. NO is known as a potent vascular smooth muscle relaxant and a regulator of cardiovascular homeostasis^22^. Endogenous endothelial NO synthase (eNOS) is a main source of synthetic NO. Previous study have been confirmed the eNOS played a significant role in vascular tone regulation. The current male eNOS knockout (KO) mice would be cardiac dysfunction and even get heart failure associated with age^23^. Meanwhile, researchers have generated mice heterozygous (+/−) or homozygous (−/−) for disruption of the eNOS gene^24^, which have been maturing applications in public research.

The main purpose of this research is to investigate the difference of the two ginseng combinations on vascular function by use of eNOS KO mice and try to get some information to achieve the maximum therapeutic response in clinic in the future. We firstly used transthoracic echocardiography (TTE) assay as a basic evaluation approach in heart and blood flow function to find the different interventions and also the difference of eNOS (−/−) and wild-type mice. Then we focused on vascular endothelial function improvement and processed the gene expression profile on thoracic aorta by Gene Chip. We hope to investigate the important differences of these commonly used ginseng drug combination in CVD and provide new knowledge regarding the compatibility of traditional herbal medicine as a basis for clinical medication.

## Results

### Protection against heart dysfunction with SFI and SMI

**Figure 1:**
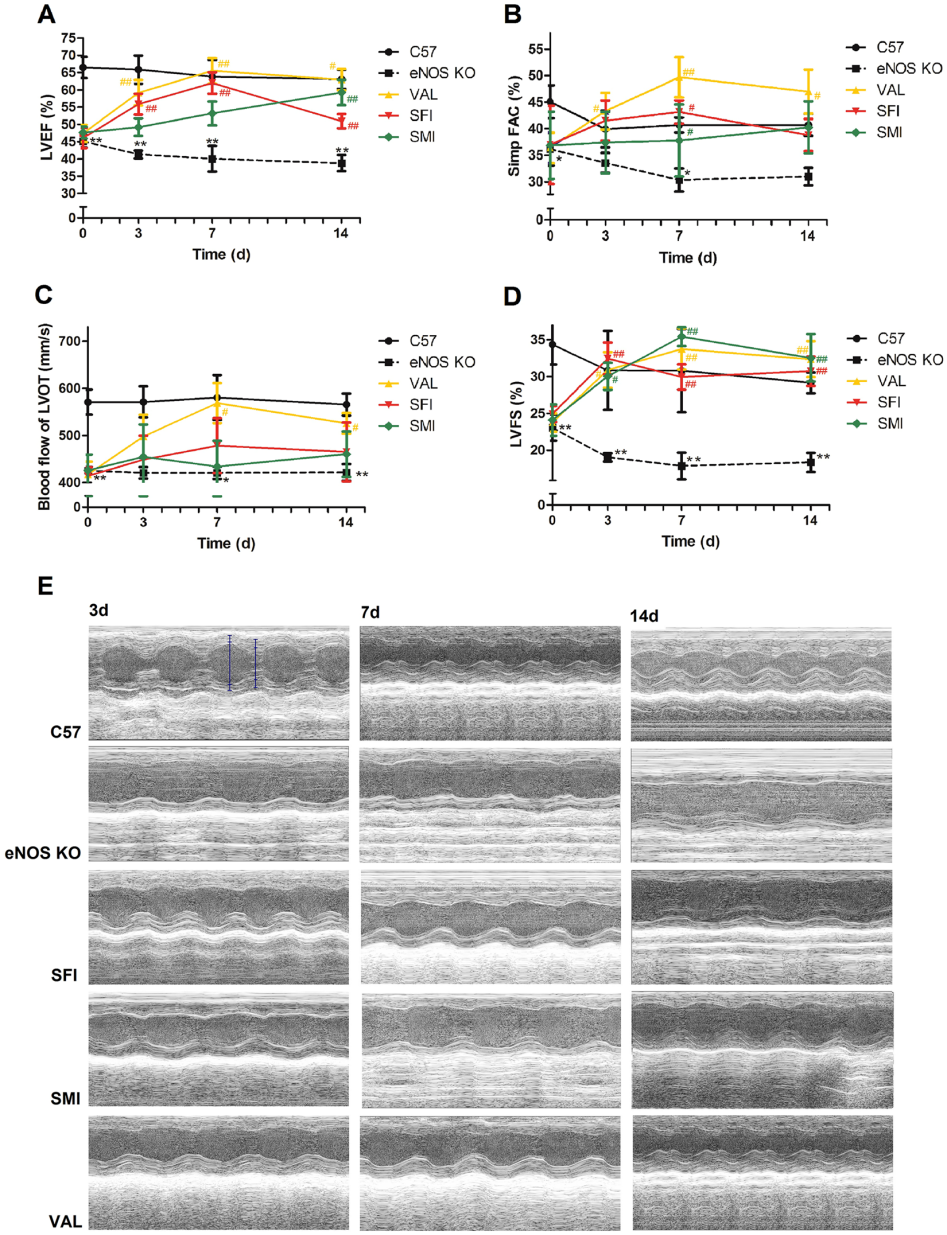
Cardiac protective effects of SFI and SMI on eNOS KO mice with echocardiography performed on days 0, 3, 7, and 14. (**A**) LVEF assay. (**B**) FAC assay. (**C**) LVOT blood flow. (**D**) LVFS assay. (**E**) Representative M-mode echocardiograms of C57, eNOS KO and treated groups of SFI, SMI and VAL, showing wall motion. The data are expressed as the mean ± SEM; n = 6 in each group; *P < 0.05, **P < 0.01 compared with C57; ^#^ *P* < 0.05, ^##^ *P* < 0.01 compared with eNOS KO.

As shown in Fig. 1, the results of the TTE assay demonstrated a time-dependent progression of heart dysfunction by gradually decreased left ventricular ejection fraction (LVEF), left ventricular fractional shortening (LVFS), fractional area change (FAC), and blood flow in the left ventricular outflow tract (LVOT) of eNOS KO mice compared to C57BL/6 J (C57). At t = 0, 3, 7 and 14d time points, LVEF presented a slight time-dependent decline in eNOS KO mice. However, no significant change was found between the different time points in KO mice. When the mice were treated with Valsartan (VAL), SFI and SMI, the decreased cardiac function was improved. The LVEF, FAC and LVOT of SFI reached their maximum values on day 7, while SMI achieved its maximum value on day 14. In addition, the LVFS of SFI was larger on day 3, while SMI was significantly larger on day 7. These findings may indicate that SFI exerted a better protective effect, especially in the early stage, while SMI was better in the chronic stage.

### The effect of SFI and SMI on blood pressure

**Figure 2:**
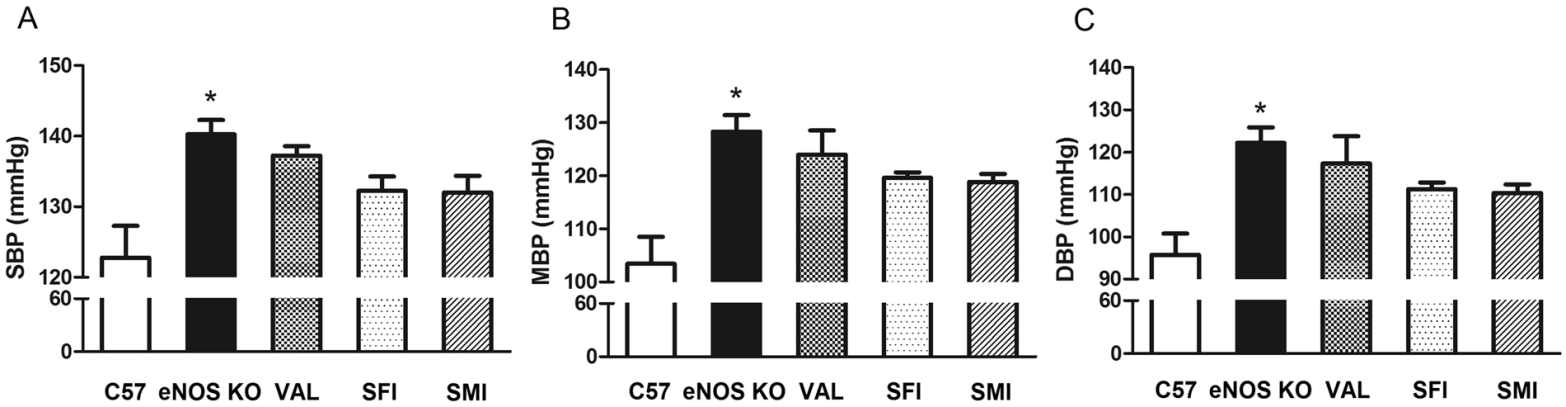
Effects of SFI and SMI on blood pressure in eNOS KO mice. (**A**) SBP of eNOS KO mice among the groups. (**B**) MBP of eNOS KO mice among the groups. (**C**) DBP of eNOS KO mice among the groups. The data are expressed as the mean ± SEM; n = 6 in each group; *P < 0.05 compared with C57.

Blood pressure was measured by the tail-cuff method at 0, 3, 7 and 14 days after drug administration. However, SFI and SMI could not decrease blood pressure in eNOS KO mice. Figure 2 shows the blood pressure values over 14 days. Compared to C57, systolic blood pressure (SBP), diastolic blood pressure (DBP) and mean blood pressure (MBP) were significantly increased in the eNOS KO group (*P* < 0.05). SFI, SMI and VAL could not detect decreases of SBP, MBP and DBP in eNOS KO mice (*P* > 0.05).

### Gene Expression Profiling of SFI and SMI in eNOS KO mice

**Figure 3:**
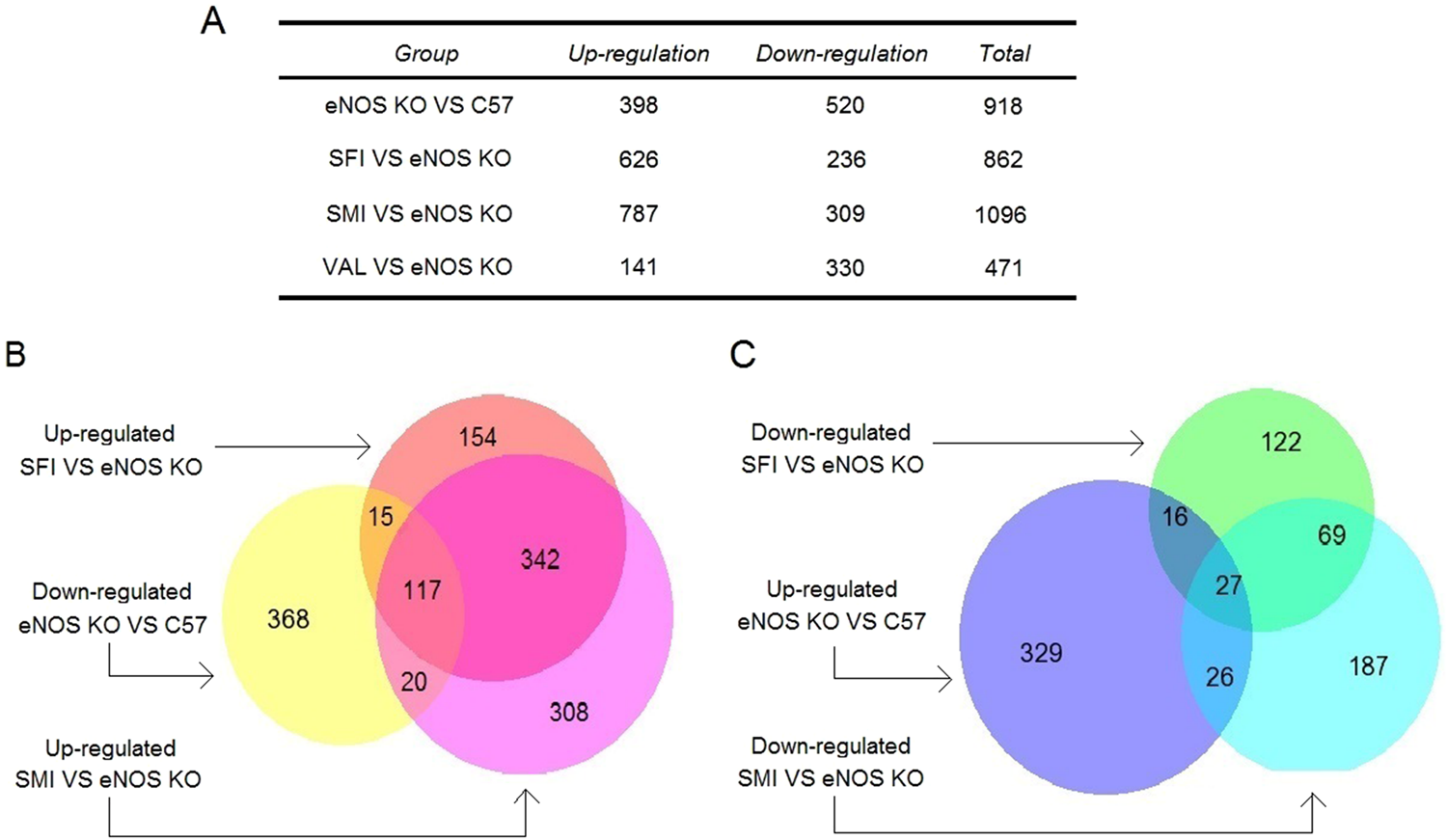
The number of differentially expressed genes. (**A**) The total differentially expressed upregulated and downregulated genes in C57, eNOS KO, SFI, SMI and VAL. (**B**) Venn diagram of overlap between upregulated genes in the SFI and SMI groups and downregulated genes in the eNOS KO group. (**C**) Venn diagram of overlap between downregulated genes in the SFI and SMI groups and upregulated genes in eNOS KO group.

Screening results identified significantly expressed genes among the SFI, SMI, eNOS KO and VAL groups, as shown in Fig. 3A. The results show a total of 918 differentially expressed genes in the eNOS KO group. A total of 862 differentially expressed genes were detected in the SFI group with 628 up-regulated genes and 234 down-regulated genes. A total of 1,096 genes were differentially expressed in the SMI group, with 787 up-regulated genes and 309 down-regulated genes. These data indicate that SMI may regulate more genes than SFI in eNOS KO mice. The distribution of this differential expression in the eNOS KO, SFI and SMI group is shown in the Venn Diagrams (Fig. 3B and C). A total of 132 and 137 SFI and SMI up-regulated genes overlapped with down-regulated genes in eNOS KO mice, respectively, while 43 and 53 down-regulated genes overlapped with up-regulated genes in eNOS KO mice, respectively. Microarray data have been submitted as Supplementary File 3.

### Gene expression bioinformatics analysis of SFI, SMI on eNOS KO mice

The Gene ID and the differences in multiple gene expressions were imported into the Ingenuity Pathway Analysis (IPA) analysis system. Diseases and biological functions, canonical pathways and regulator effect networks related to differential genes were analysed.

**Figure 4:**
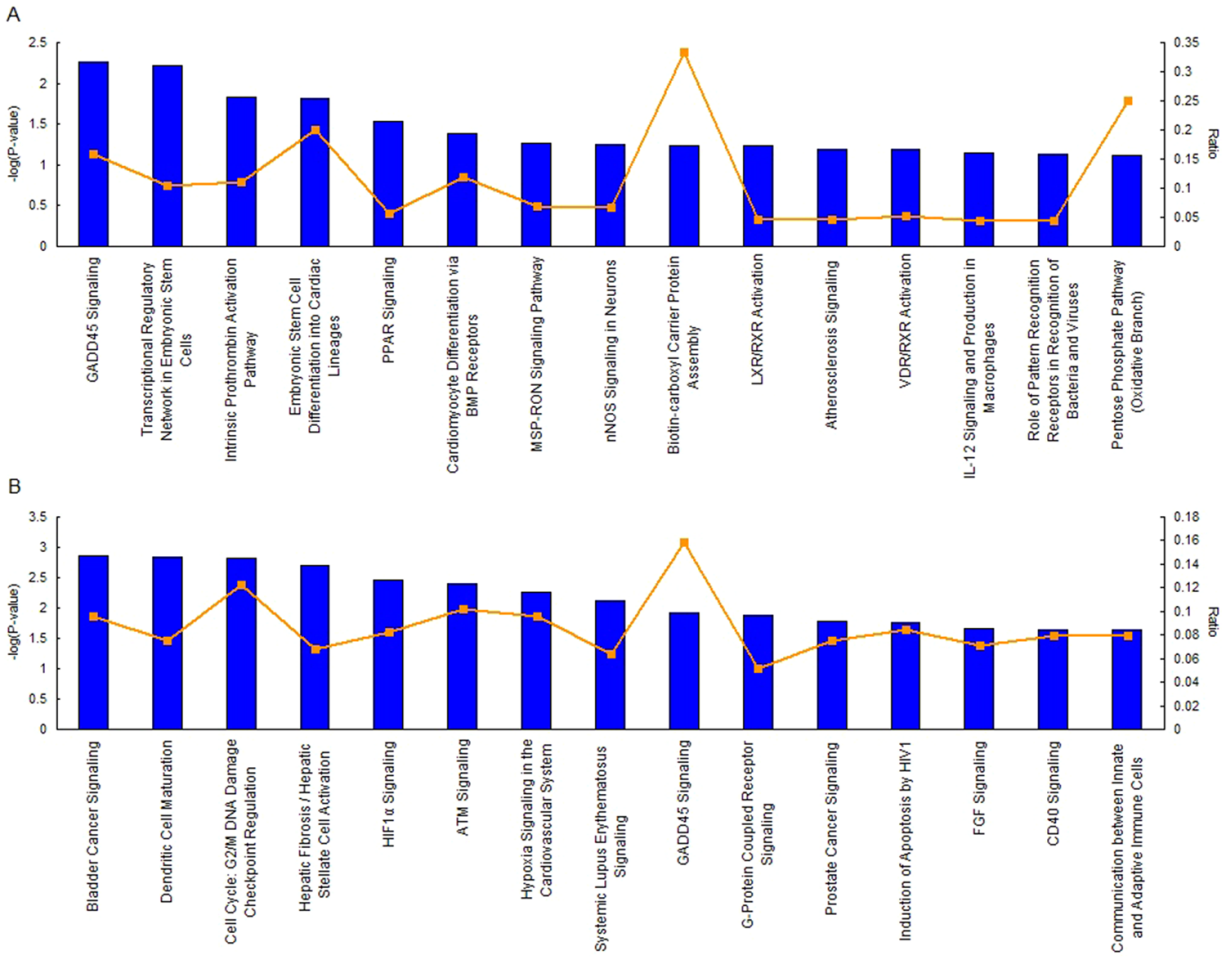
SFI and SMI canonical pathways obtained by IPA. (**A**) Top 15 canonical pathways in the SFI group according to IPA. (**B**) Top 15 canonical pathways in the SMI group according to IPA. Blue bars indicate −log(P-value), while yellow points denote the ratio of genes.

The canonical pathways of SFI and SMI are shown in Fig. 4. The figures only show the top 15 pathways according to *P*-value. The significant pathways included GADD45 signaling, NF-κB signaling, protein kinase A signaling and cAMP-mediated signaling. Of all the main pathways, SFI also could regulate PPAR signaling (TOP 5, −log(p-value) = 1.53E + 00), cardiomyocyte differentiation via BMP receptors (TOP 6,−log(p-value) = 1.38E + 00) and VDR/RXR activation (TOP 12, −log(p-value) = 1.19E + 00). HIF1α Signaling (TOP 5, −log(p-value) = 2.46E + 00), ATM signaling (TOP6, −log(p-value) = 2.41E + 00), G-Protein, coupled receptor signaling (TOP 6, −log(p-value) = 1.88E + 00) and FGF signaling pathway (TOP 13, −log(p-value) = 1.66E + 00) were the main pathways that regulated by SMI. The whole pathway list was showed in Supplementary File 1.

**Figure 5:**
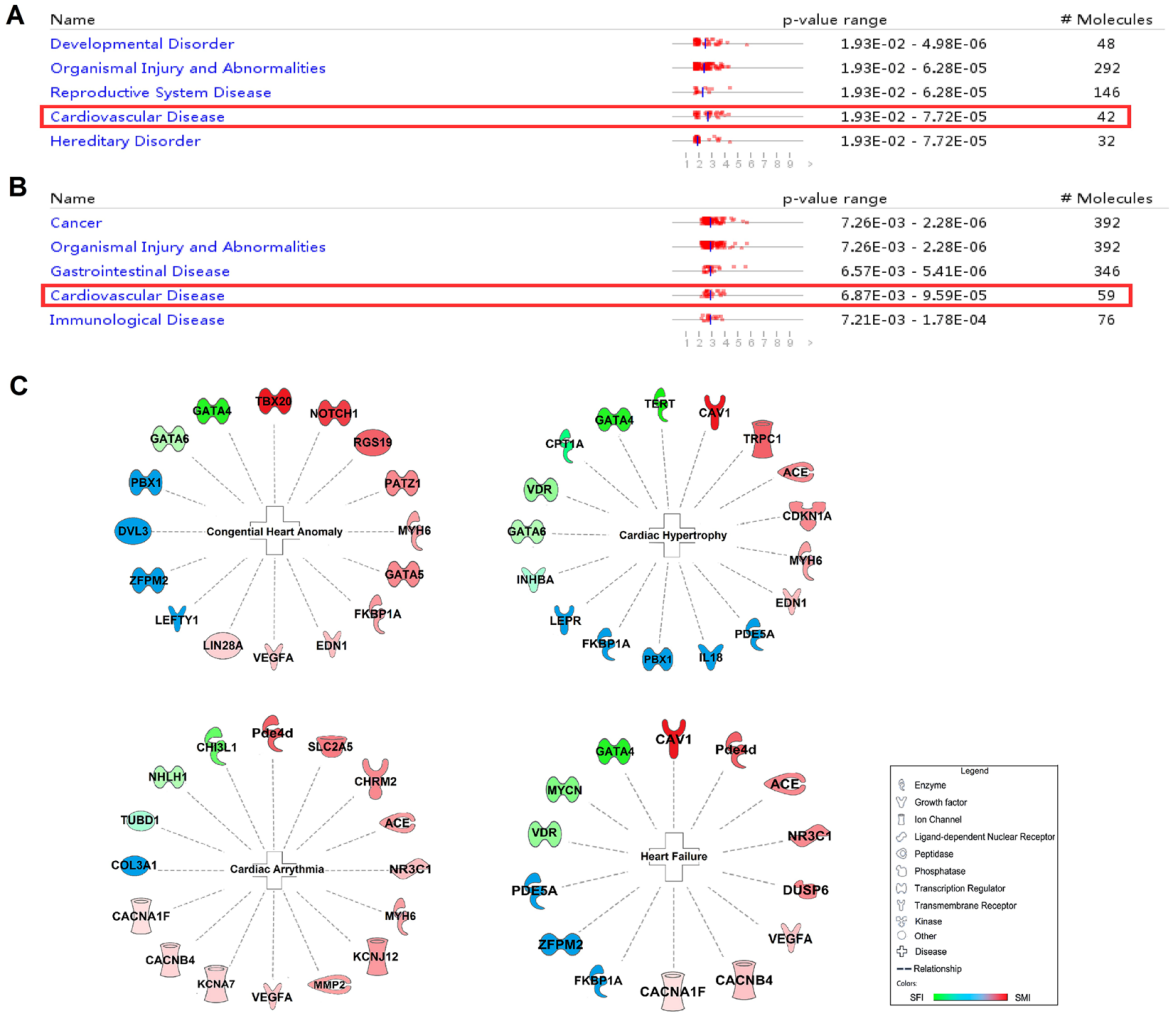
Summary of the diseases, disorders and the significant molecules of the four diseases identified by IPA. (**A**) The list of top 5 diseases and disorders with their respective scores obtained from IPA in the SFI group. (**B**) The list of top 5 diseases and disorders with their respective scores obtained from IPA in the SMI group. (**C**) a: The significant congenital heart molecular profile identified by IPA. b: The significant cardiac hypertrophy molecular profile identified by IPA. c: The significant cardiac arrhythmia molecular profile identified by IPA. d: The significant HF molecular prolife identified by IPA. (The regulated SFI molecules are shown in green, while those associated with SMI are shown in red; common molecules are shown in blue.)

**Figure 6:**
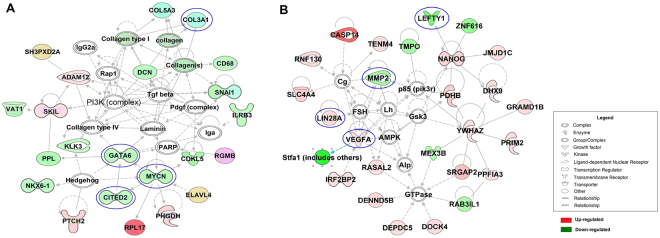
The most significant molecular networks identified by IPA. (**A**) The most significant SFI molecular network as identified by IPA. (**B**) The most significant SMI molecular networks as identified by IPA.

A comparison of diseases and disorders with SFI and SMI are shown in Fig. 5. Both medicines had an impact on pathways associated with CVD and organismal injury and abnormalities. Meanwhile, SFI also influenced developmental disorder, reproductive system disease and hereditary disorder. SMI worked up to a point with cancer, gastrointestinal disease and immunological disease. In CVD, the IPA demonstrated that for both the SFI and SMI groups more than 65 percent of the diseases and functions were congenital heart anomaly, cardiac hypertrophy, cardiac arrhythmia and HF. Using with IPA analysis, we obtained molecular information of the four diseases, as shown in Fig. 6. Based on this data, GATA4 (GATA binding protein 4), GATA6 (GATA binding protein 6), MYCN (v-myc avian myelocytomatosis viral oncogene neuroblastoma derived homolog) and COL3A1 (collagen, type III, alpha 1) may play key roles in SFI-mediated CVD improvement. In contrast, VEGFA (vascular endothelial growth factor A), CAV1 (caveolin 1, caveolae protein), MMP2 (matrix metallopeptidase 2) and ACE (angiotensin I converting enzyme) were associated with SMI. In addition, IL-18 (interleukin 18), PDE5A (phosphodiesterase 5 A, cGMP-specific), and PBX1 (pre-B-cell leukemia homeobox 1) were common among both treatments. The whole diseases list was showed in Supplementary File 1.

To predict the participation of other interacting molecules in the pathways, we performed IPA gene networks. Figure 6 shows the most remarkable networks related to CVD and SFI and SMI treatment. A few genes previously shown to be important were found in these top networks, such as GATA6, MYCN, COL3A1 and PI3K (complex) in SFI, and VEGFA, MMP2 and ACE in SMI.

### Validation of Microarray Analysis

**Figure 7:**
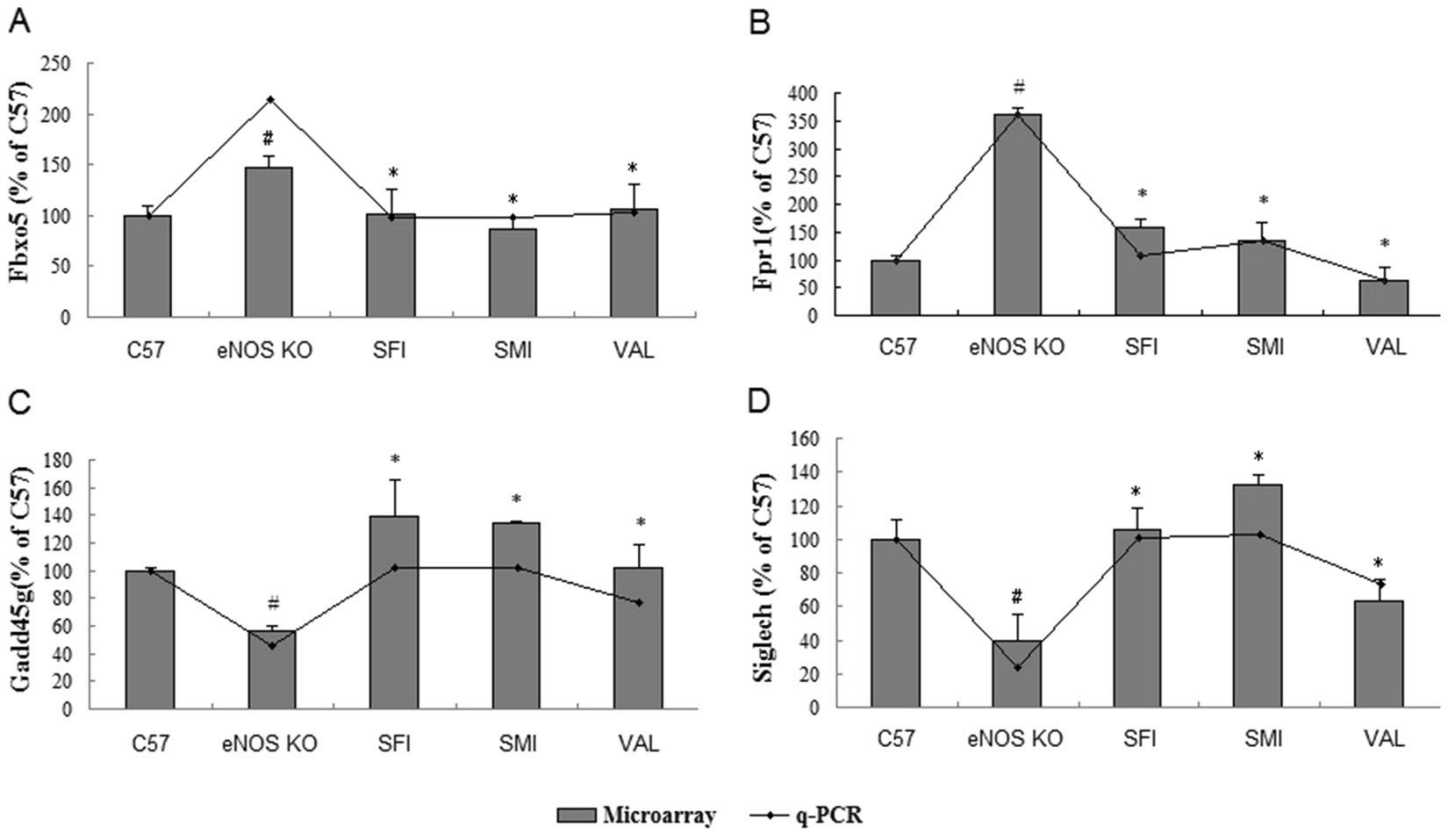
Validation of microarray data by qPCR. (**A**) Fbxo5 gene analysis. (**B**) Fpr1 gene analysis. (**C**) Gadd45g gene analysis. (**D**) Siglech gene analysis. The data are expressed as the mean ± SEM; n = 3 in each group; *P < 0.05, **P < 0.01 compared to C57; ^#^ *P* < 0.05, ^##^ *P* < 0.01 compared to eNOS KO mice.

In Fig. 7, validation of the microarray results was performed using quantitative real-time polymerase chain reaction (qPCR) on a subset of the genes identified. The Fbxo5 (F-box protein 5) and Fpr1 (formyl peptide receptor 1) genes were upregulated significantly in the eNOS KO group compared to the C57 group, and SFI, SMI and VAL could downregulate these genes. In addition, Gadd45g (the growth arrest and DNA-damage-inducible, gamma) and Siglech (sialic acid binding Ig-like lectin H) genes were downregulated in eNOS KO mice, and the tendencies were reversed by SFI, SMI and VAL. Generally, there was good correlation in directionality between the two techniques.

## Discussion

In this study we investigated the similarities and differences between SFI and SMI on eNOS KO mice to understand the effects of combining ginseng with other herbal medicines. TTE showed that both SMI and SFI could improve the decreased cardiac function of eNOS KO mice by increasing LVEF, LVFS, FAC and LVOT. However, we found that SFI (ginseng combined with aconiti lateralis) might be more efficient in the early stage (3–7d) of CVD, whereas SMI (ginseng combined with ophiopogonis) was more impactful in the chronic stage (7–14d). The result is just coincided with our previous systematic review and meta-analysis^21^. Several studies have reported that there are some differences about SFI and SMI in the treatment of CVD. For example, SFI could improve cardiac function and myocardial oxidative stress by increasing left ventricular systolic pressure (LVSP), left ventricular end diastolic pressure (LVEDP) and superoxide dismutase (SOD) after six hours of administration^25^. In addition, SFI had a positive inotropic effect on myocardial cells and it combined nitroglycerine and furosemide with acute left ventricular failures was significant after 30 minutes of treatment. Moreover, it was previously reported that SFI could restore the ability of Na^+^-K^+^-ATPase and Ca^2+^-ATPase enzyme activities at 12-min on myocardial metabolism during ventricular fibrillation (VF)^26,27^. It also had a faster therapeutic effect. Combined use of SFI and early goal-directed therapy (EGDT) on septic shock patients, heart rate decreased at 24, 48, and 72 h; while gamma-glutamyl transpeptidase and glutamate oxaloacetate transaminase levels increased at one day^28^. On the other hand, SMI could significantly decrease the level of TNF-α and IL-6 at 24 hours in post-cardiac arrest syndrome^29^. A large number of clinical trials have shown that SMI could benefit the patients with chronic cor pulmonale HF^14^. At the same time, patients with coronary heart disease could get an increase the number of endothelial progenitors cells (EPCs) after SMI treatment by 7 to 14 days^30^. After 2 weeks of SMI treatment, cardiac function, such as stroke volume, cardiac index, was significantly improved^31^. All these results indicated that SFI provides a better effect in the early stage while SMI provides a better effect in the chronic stage of CVD. The different efficacy of SFI and SMI might related to the different regulated genes on vascular function according to the current research.

Previous reports have reported that ginsenoside’s cardiac protection effect was partially via promoting releasing NO from endothelium^32^. Aconiti lateralis could inhibit cardiomyocyte apoptosis and myocardial damage and also modulate heart rate, rhythm and hemodynamics^33,34^. Ophiopogonis can alleviate ischemia/reperfusion injury in isolated myocardium^35^. A clinical trail showed that SFI could reduce the myocardial injury^36^. Its effects on cardiac performance and coronary circulation are mediated by ginseng and aconiti lateralis via NO release, increased coronary flow, left ventricular developed pressure (LVDP) and the rate-pressure product (RPP)^37^. Meanwhile, SFI had a reduced inflammatory reaction in patients with acute myocardial infarction complicated by cardiac shock^38^. At the same time, SMI is used for treatment of chronic HF^39,40^. Moreover, it can alleviate the lung injury after cardiac pulmonary bypass through raise the level of NO and reduce the level of ET-1 (endothelin-1)^41^, and improve the endothelial function in patients with coronary heart disease complicated with diabetes mellitus^42^.

Evidences have shown the worthy to a deep research with NO related mechanism of SFI and SMI. By Agilent Mouse Gene Expression and IPA we analyzed the different genes and pathways of the two interventions. To determine major treatment features of CVD, we used the “diseases and functions” sections of the IPA software and selected the top 4 significant correlation diseases according to score. The results demonstrated that congenital heart anomaly, cardiac hypertrophy, cardiac arrhythmia and HF account for a large percentage in SMI group. To predict correlations of relevant genes and pathways, “networks” of IPA were scored based on the molecules contained in these networks. The IPA analysis showed that GATA4 and GATA6 were significantly down-regulated in SFI at −2.5 and −2.4-fold, respectively, when treating CVD. GATA4 and GATA6 are important transcription factors that primarily impact myocardial cell proliferation, differentiation, and apoptosis^43,44^. Previous studies showed that GATA4 appears to be essential and its upregulation is sufficient to promote myocyte survival, in part through transcriptional regulation of the anti-apoptotic Bcl-2 gene^45^. Similarly, GATA4 is a key nuclear effecter in numerous signalling pathways that are activated by hormones and growth factors that cause myocyte enlargement and cardiac hypertrophy^46^. Meanwhile, the primary function of GATA-6 during cardiovascular development is to regulate morphogenetic patterning of the cardiac outflow tract and aortic arch^47^. Both GATA4 and GATA6 can act directly on myocardial cells and the cardiac outflow tract to improve heart function in eNOS KO mice.

The SFI molecular network analysis (Fig. 6A) showed that Collagen type III accounted for a large proportion. COL3A1 was the primary down-regulated gene in this network at −2.4-fold. COL3A1 is a member of the collagen family and is primarily expressed in extensible connective tissues, including skin and vessels^48^. COL3A1 is essential for normal collagen I fibrillogenesis in the cardiovascular system^49^. Collagen type III is the second most abundant collagen in human tissues after collagen type I and is expressed in blood vessels. It is encoded by the COL3A1 gene and is closely linked with the COL5A2 (collagen, type V, alpha 2) gene^50^. The result demonstrated that SFI directly improved myocardial function to treat CVD via important genes, such as GATA4, GATA6 and COL3A1. These results also partly explained why the effect of SFI was more pronounced in the early stage in the TTE assay.

The IPA molecular network showed that VEGFA was the vital component and was up-regulated by SMI at 2.3-fold compare to eNOS KO mouse. VEGFA is one of the most potent inducers of angiogenesis and is potent and specific for vascular endothelial cells^51^. Blocking VEGF-A may lead to endothelial dysfunction and adverse vascular effects^52^. VEGFA closely correlated with coronary artery disease^53^, atherosclerosis^54^, and HF^55^. Meanwhile, the IPA molecular analysis of SMI indicated that ACE was also an importantly down-regulated gene at −2.2-fold. The main site of ACE expression is the vascular endothelium, underlining its vital role in normal blood pressure control^56^. Both VEGFA and ACE are closely related to endothelial cells, which can respond with appropriate control and regulatory processes to maintain homeostasis. Such responses can include the release of paracrine mediators, such as NO, prostacyclin and ET-1^57–59^. In brief, both VEGFA and ACE defend against heart dysfunction by protecting endothelial cells, which can provide nutrients and promote the development of myocardial cells.

In addition, we found that MMP2, a zinc dependent protease, played a key role in SMI and is down-regulated at −2.3-fold^60^. MMP2 participates in many biological processes, such as inflammation, cardiomyocyte hypertrophy and systolic HF^61,62^. In addition, it can also modulate signalling of growth factors, such as VEGF (vascular endothelial growth factors)^63^. The common thing in these genes is that they have close correlation with endothelial cells from these evidence. Meanwhile, there is no clinic evidence showed SMI’s effect on myocardium. Therefore, SMI may be not directly involved in myocardial cells, instead, it relies on the effects of endothelial cells. This also confirms the results from TTE, which demonstrated that SMI was more involved in long-term treatment than SFI. This may be related to MMP2 and the protection of endothelial cells by VEGFA and ACE.

Inflammation has been well known to play an important role in CVD development and progress. IL-18, a pro-inflammatory cytokine in the interleukin 1 (IL-1) cytokine superfamily, is a common down-regulated gene in SFI and SMI at −2.1 and −2.0-fold, respectively, compared to eNOS KO mice. IL-18 plays an important role in immune, infectious, and inflammatory diseases due to its induction of IFN-gamma^64^. The “canonical pathways” section of the IPA analysis showed that IL18 was primarily involved with PPAR signaling (−log(p-value) = 1.53E + 00) in SFI, and NF-κB signaling and IL-12 signaling and production in macrophages (−log(p-value) = 1.64E + 00 and 1.50E + 00, respectively) in SMI.

From the top 15 canonical pathways by IPA analysis (Fig. 4), it is worth mentioning that Gadd45 signaling is the only common pathway in SFI and SMI (−log(p-value) = 2.26E + 00, −log(p-value) = 1.92E + 00, respectively). Gadd45 play important roles in cellular genotoxic and non-genotoxic stress responses acting as stress sensors and tumor suppressors, which are rapidly induced after DNA damage, resulting in cell cycle arrest and/or apoptosis, DNA repair mechanisms^65,66^. It have been implicated in linking NF-κB and MAPK, such as involved in the activity of NF-κB in the cell death and survival control^67,68^. Previous studies have also shown that non-steroidal anti-inflammatory drugs (NSAIDS) rely on Gadd45 up-regulation for induction of cell cycle arrest and apoptosis in tumor cells^69^. Gadd45 signaling is closely related to inflammation. Our microarray data showed that SFI and SMI could up-regulated Gadd45g at 2.6 and 2.4-fold, respectively, compared to eNOS KO mice. In addition, we used qPCR to verified Gadd45g and the result was coincided with microarray data (Fig. 7C). These findings may provide a theoretical basis for clinical medication.

**Figure 8:**
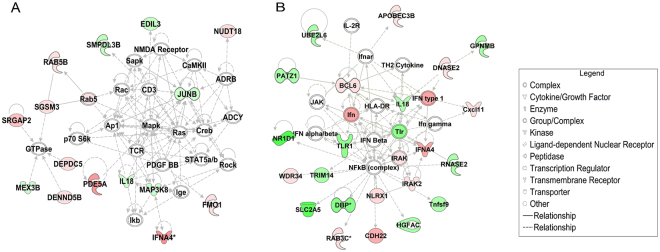
The most significant inflammation networks identified by IPA. (**A**) The most significant inflammation network of SFI identified by IPA. (**B**) The most significant inflammation network of SMI identified by IPA.

By whole genome analysis, we found oxidative stress, inflammation, and apoptosis were all involved in eNOS KO induced injury. The pathogenesis is too complex for the classic single drug treatment. Herbal combination should be paid attention to especially on enhance curative effects and expand therapeutic range to adapt to complex disease. In this study, there are some similarities as well as some differences in the two ginseng combinations. Simultaneously, we should noticed the advantage of multi targets by drug combination in progression of complex diseases (Fig. 8). The appropriate adjustment of drug combination could lead to a better accurate medical care in clinic.

In addition to the result, there is an abnormal phenomena that we observed. Figure 1A showed LVEF had the significant drop on day 14 in SFI group. This may be related to a potential problem caused by the alkaloids from Aconiti lateralis with prolonged the course of treatment^70–72^. Therefore, in general, SFI have a protective effect on the heart in short-term intervention in clinic. However, from the current results, the feasibility and safety to use SFI for 14 days is worth deep research Fig. 8.

In conclusion, our study has demonstrated that ginseng combined with aconiti lateralis was more efficient in the early stage compared to ginseng and ophiopogonis, which was more impactful in the chronic stage. From the result of TTE assay and IPA analysis, it suggested that SFI and SMI could both improve the LVEF and modulate Gadd45 Signaling from TOP15 canonical pathways. The distinction was that SFI showed a better effect in the early treatment stage, manifested in improving LEVF and FS, and might directly improved myocardial function to treat CVD via GATA4, GATA6 and COL3A1. While SMI exerted better protective effects at the chronic stage, which may be related to MMP2 and endothelial cell protection by VEGFA and ACE. By *in vivo* experiment and gene expression profiles, the results indicated that SFI could provide a better effect in the early stage while SMI presented a better effect in the late stage of heart dysfunction. The result could partly show the value to adjust herbal combination in the precision medication in clinic.

## Materials and Methods

### Animals

Wild-type (C57) and eNOS KO mice (B6.129P2-Nos3tm1Unc/NJU) (Six-to-eight weeks) weighing 18–22 g were purchased from the National Resource Center of Model Mice (Nanjing, China. Permit NO:SCXK (Su 2010-0001)). All animals were fed a standard feed and provided water ad libitum while housed in the Institute of Radiation Medicine Chinese Academy of Medical Sciences (Tianjin, China). All experiments were performed under the Guidelines for Animal Experiments at the Tianjin University of Traditional Chinese Medicine. The Animal Ethics Committees of the Faculty of Medicine approved all experimental protocols in accordance with the Principles of Laboratory Animal Care and Use in Research (Permit Number: SCXK 2010-0001).

### Drug intervention

SFI (Yaan Sanjiu Pharmaceutical CO.LTD, Sichuan, China) and SMI (Chiatai Qingchunbao Pharmaceutical CO.LTD, Jiangsu, China) were administered via a single intraperitoneal dose of clinical equivalence 0.216 g/kg and 0.144 g/kg daily. Meanwhile, C57 mice, serving as the control, and eNOS KO mice, as the model, were given intraperitoneal injections of equal volumes of saline. VAL (10.4 mg/kg, daily, Novartis, Beijing, China) were administered intragastrically. SFI, SMI, VAL and saline were injected for 2 weeks.

### TTE assay

The Vevo 2100 ultrasound system (VisualSonics, Toronto, Canada) was turned on standby, and the MS400 probe adjusted to a suitable position. Isoflurane (Martx, New York, USA) was used for anaesthesia (totally anaesthesia: 1% O_2_ + 5% isoflurane; continued anaesthesia: 1% O_2_ + 2% isoflurane). Mice were fixed supinely and coupling agent was smeared after depilation. The FAC was evaluated in B-Mode, the LVEF and LVFS were obtained in M-Mode, and blood flow in the LVOT was measured in Colour Doppler Mode. All measurements were performed at baseline and 3, 7, and 14 days after drug administration.

### Blood pressure measurement

Blood pressure was measured by the tail-cuff method (BP98AWU, Softron Biotechnology, Tokyo, Japan) at 0, 3,7 and 14 days after drug administration. The SBP, DBP and MBP values were continuously monitored and recorded. Two measurements were obtained by an investigator-blinded method and averaged for each mouse.

### Microarray Analysis

The thoracic aortas were excised from the mice. The peri-adventitial, fibro-adipose tissues were carefully isolated. Total RNA was extracted using mirVana RNA Isolation Kit (Thermo Scientific, Waltham, USA), and quantified using the NanoDrop ND-2000 (Thermo Scientific, Waltham, USA) and the RNA integrity was evaluated using Agilent Bioanalyser 2100 (Agilent Technologies, Palo Alto, USA). Sample labelling, microarray hybridization and washing were successively performed according to the manufacturer’s instructions. The Agilent SurePrint G3 Mouse Gene Expression (8*60 K, Design ID: 028005) was used in this experiment. Briefly, after transcribing total RNA to double strand cDNA, cDNA were synthesized into cRNA and labelled with Cyanine-3-CTP. Then, we hybridized the labelled cRNAs onto the microarray and washed. The arrays were scanned by the Agilent Scanner G2505C (Agilent Technologies).

Array images were analysed using Feature Extraction software (version10.7.1.1, Agilent Technologies) to obtain raw data. Genespring were assessed with the raw data as a basic analysis. The raw data were normalized with the quantile algorithm. Among the conditions, one probe that was flagged as “Detected” with 100% of the values in at least one group was chosen for further data analysis. Differentially expressed genes were then identified through fold change as well as *P* value calculated with t-test. The threshold set for up- and down-regulated genes was a fold change > = 2.0 and a *P* value < = 0.05, these genes which satisfy the set threshold had been collected as “Differential genes” for IPA (Ingenuity Systems, Redwood City, CA, USA, www.ingenuity.com) analysis, which is supported by Ingenuity Knowledge Base. Canonical pathways, diseases and biological functions and regulator effect networks related to differential genes were generated by the software algorithm. A fold change higher than 2 means “activated” while lower than −2 means “inhibited”. Statistical significance was set at *P* < = 0.05. The “canonical pathways” section was calculated using the right-tailed Fisher Exact Test. We choose the top 15 pathways to analyse. Blue bars indicate −log (*P*-value), while yellow points denote the ratio of genes. The meaning of the ratio of genes is, in a specified path, all the genes contained in this pathway (currently confirmed) is assumed to be A, and the differences expression genes involved in experimental group (assuming E). The ratio of genes = E/A *100%. It vividly shows the enrichment state of the group of genes in this pathway. To determine major treatment features of CVD, we used the “diseases and functions” sections of the IPA software and selected the top 4 significant correlation diseases according to score. To predict correlations of relevant genes and pathways, “networks” of IPA were scored based on the molecules contained in these networks.

### Validation of Microarray results by qPCR

Total RNA was extracted from aorta using Trizol reagent (Invitrogen, Chicago, USA). The purity of RNA was verified by calculating the absorbance ratio of 1.8–2.0 at 260/280 nm, and the RNA concentration was quantified spectrophotometrically at 260 nm (Malcom, Tokyo, Japan). Total cellular RNA (2.0 μg) was reverse transcribed by TaqMan Reverse Transcription Reagents (Applied Biosystems, Foster City, USA) and the cDNA was used in qPCR reactions. The primers (Sangon Biotech, Shanghai, China) used were as follow: β-actin F: 5′-AGAGGGAAATCGTGCGTGAC-3′; R: 5′-CAATAGTGATGACCTGGCCGT-3′; Fbxo5 F: 5′-GCGCCTTTTAAGAGCTGCGG-3′; R: 5′-GCTCATGCCGCAAAACTCG-3′; Fpr1 F: 5′-CCTTGGCTTTCTTCAACAGC-3′; R: 5′-GCCCGTTCTTTACATTGCAT-3′; Gadd45g F: 5′-CGCACAATGACTCTGGAAGA-3′; R: 5′-CAGGGTCCACATTCAGGACT-3′; Siglech F: 5′-AGACACTGGAGCTTGGTCGT-3′; R: 5′-CCTGACAGGTGAGGTTGGTT-3′. The amplification was performed in an ABI 7500 Real-Time PCR System (Applied Biosystems, Foster City, USA). β-actin mRNA was measured as an internal control and the expression of related target genes was determined by the 2^−ΔΔCT^ method.

### Statistical analyses

Statistical analysis was conducted using SPSS 16.0. The results were represented as the means ± standard error of the mean (SEM), and all data passed a normality test. Statistical significance was assessed by one-way analysis of variance (ANOVA) followed by Tukey’s test. A *P* value < 0.05 was considered statistically significant for all cases.

## Electronic supplementary material

Supplementary File 1

Supplementary File 2

Supplementary File 3
