# Supplementary material for: Heart function and thoracic aorta gene expression profiling studies of ginseng combined with different herbal medicines in eNOS knockout mice
Source: Sci Rep. 2017 Nov 13;7:15431. doi: 10.1038/s41598-017-15819-2 (PMC5684410; doi:10.1038/s41598-017-15819-2)
Supplement: Supplementary file 1 [file 41598_2017_15819_MOESM1_ESM.doc]

**Supplementary File 1**

**Heart function and thoracic aorta gene expression profiling studies of ginseng combined with different herbal medicines in eNOS knockout mice.**

**Yuchen Qian^1^,** ^+^**, Pan Li^1^,**^+^**, Bin Lv^1^, Xiaoqing Jiang^1^, Ting Wang^1^, Han Zhang^1^, Xiaoying Wang^1, 2, *^, Xiumei Gao^1^**

^1^State Key Laboratory of Modern Chinese Medicine, Tianjin University of Traditional Chinese Medicine, Tianjin, 300193, China.

^2^College of Traditional Chinese Medicine, Tianjin University of Traditional Chinese Medicine, Tianjin, 300193, China.

[***Correspondence:**](mailto:*wxy@tjutcm.edu.cn)

[Wang, Xiaoying M.D.](mailto:*wxy@tjutcm.edu.cn)

[Address: 312 Anshanxi Road,Nankai District,Tianjin,P.R.China,300193](mailto:*wxy@tjutcm.edu.cn)

[E-mail:wxy@tjutcm.edu.cn](mailto:*wxy@tjutcm.edu.cn)

**Table S1 MS/MS data in (+/-) ESI modes and the identification results for the bioactive compounds of SMI.**

| **Peak No.** | **RT**  **(min)** | **M/Z** | **Mode** | **MS-MS** | **Composition** | **Identiﬁcation** |
| --- | --- | --- | --- | --- | --- | --- |
| 1 | 9.310 | 932.5327 | Neg/Pos | 931[M-H]^-^,637[M-H-Ara(p)-Glc]^-^,475[M-H-Ara(p)-2Glc]^-^ | C_47_H_80_O_8_ | Notoginsenoside R1 |
| 2 | 9.774 | 800.4920 | Neg/Pos | 799[M-H]^-^,637[M-H-Glc]^-^,475[M-H-2Glc]^-^ | C_42_H_72_O_14_ | Ginsenosides Rg1 |
| 3 | 9.823 | 946.4834 | Neg/Pos | 945[M-H]^-^,783[M-H-Glc]^-^,637[M-H-Glc-Rha]^-^ | C_48_H_82_O_18_ | Ginsenosides Re |
| 4 | 10.061 | 478.2230 | Neg/Pos | 477[M-H]^-^,459[M-H-H_2_O]^-^,315[M-H-Glc]^-^ | C_22_H_38_O_11_ | aplosy(1—6)  glucosamine bomeo |
| 5 | 12.916 | 800.4947 | Neg/Pos | 799[M-H]^-^,637[M-H-Glc]^-^,475[M-H-2Glc]^-^ | C_42_H_72_O_14_ | Ginsenosides Rf |
| 6 | 13.470 | 770.4785 | Neg/Pos | 769[M-H]^-^,637[M-H-Ara(p)]^-^,475[M-H-Ara(p)- Glc]^-^ | C_41_H_70_O_13_ | Notoginsenoside R2 |
| 7 | 14.104 | 784.4964 | Neg/Pos | 783[M-H]^-^,621[M-H-Glc]^-^,459[M-H-2Glc]^-^ | C_42_H_72_O_13_ | Ginsenosides Rg2 |
| 8 | 14.257 | 1108.5959 | Neg/Pos | 1107[M-H]^-^,945[M-H-Glc]^-^,783[M-H-2Glc]^-^ | C_54_H_92_O_23_ | Ginsenosides Rb1 |
| 9 | 14.760 | 1078.5916 | Neg/Pos | 1077[M-H]^-^,945[M-H-Ara(p)]^-^,915[M-H-Glc]^-^ | C_53_H_90_O_22_ | Ginsenosides Rc |
| 10 | 14.920 | 956.4962 | Neg/Pos | 955[M-H]^-^,937[M-H-H_2_O]^-^,793[M-H-Glc]^-^, | C_48_H_76_O_19_ | Ginsenosides Ro |
| 11 | 15.339 | 1078.5920 | Neg/Pos | 1077[M-H]^-^,945[M-H-Ara(p)]^-^,783[M-H- Ara(p)]^-^ | C_53_H_90_O_22_ | Ginsenosides Rb2 |
| 12 | 15.518 | 1078.5848 | Neg/Pos | 1077[M-H]^-^,915[M-H-Glc]^-^,783[M-H- Ara(p)]^-^ | C_53_H_90_O_22_ | Ginsenosides Rb3 |
| 13 | 16.460 | 946.5469 | Neg/Pos | 945[M-H]^-^,783[M-H- Glc]^-^,621[M-H-2Glc]^-^ | C_48_H_82_O_18_ | Ginsenosides Rd |
| 14 | 18.788 | 870.4498 | Neg | 869[M-H]^-^,851[M-H-H_2_O]^-^,737[M-H-Ara(p)]^-^ | C_44_H_70_O_17_ | Spirost-5-ene-3,17-diol,(3β,17αOH,25R)-from,  3-O-[α-L-rhamnopyranosyl-(1-2)-α-L-arabinofuranosyl-  (1-4)-β-D-glucopyranoside |
| 15 | 21.053 | 784.4973 | Neg/Pos | 783[M-H]^-^,621[M-H-2Glc]^-^,829[M-H+HCOOH]^-^ | C_42_H_72_O_13_ | iso-Ginsenosides Rg3 |
| 16 | 21.390 | 784.4973 | Neg/Pos | 783[M-H]^-^,621[M-H-2Glc]^-^,829[M-H+HCOOH]^-^ | C_42_H_72_O_13_ | Ginsenosides Rg3 |
| 17 | 25.071 | 766.4789 | Neg/Pos | 765[M-H]^-^,603[M-H- Glc]^-^,441[M-H-2Glc]^-^ | C_42_H_70_O_12_ | Ginsenosides Rk1 |

**Table S2The Chromatographic conditions of UPLC analysis.**

| Time (min) | Flow ( mL/min) | Rate %A (water-0.1% Formic acid) | Rate %B (Acetonitrile) |
| --- | --- | --- | --- |
| 0 | 0.4 | 98.0 | 2.0 |
| 10 | 0.4 | 73.0 | 27.0 |
| 15 | 0.4 | 65.0 | 35.0 |
| 17 | 0.4 | 60.0 | 40.0 |
| 22 | 0.4 | 50.0 | 50.0 |
| 27 | 0.4 | 38.0 | 62.0 |
| 29 | 0.4 | 30.0 | 70.0 |
| 31 | 0.4 | 0.0 | 100.0 |
| 32 | 0.4 | 0.0 | 100.0 |
| 33 | 0.4 | 98.0 | 2.0 |
| 35 | 0.4 | 98.0 | 2.0 |

**Table S3 Canonical pathways of SFI VS eNOS KO.**

| **Ingenuity Canonical Pathways** | **-log(p-value)** | **Ratio** |
| --- | --- | --- |
| GADD45 Signaling | 2.26E+00 | 1.58E-01 |
| Transcriptional Regulatory Network in Embryonic Stem Cells | 2.22E+00 | 1.05E-01 |
| Intrinsic Prothrombin Activation Pathway | 1.83E+00 | 1.11E-01 |
| Embryonic Stem Cell Differentiation into Cardiac Lineages | 1.82E+00 | 2.00E-01 |
| L-dopachrome Biosynthesis | 1.71E+00 | 1.00E+00 |
| PPAR Signaling | 1.53E+00 | 5.62E-02 |
| Cardiomyocyte Differentiation via BMP Receptors | 1.38E+00 | 1.18E-01 |
| MSP-RON Signaling Pathway | 1.27E+00 | 6.82E-02 |
| nNOS Signaling in Neurons | 1.25E+00 | 6.67E-02 |
| Biotin-carboxyl Carrier Protein Assembly | 1.24E+00 | 3.33E-01 |
| LXR/RXR Activation | 1.23E+00 | 4.63E-02 |
| Atherosclerosis Signaling | 1.19E+00 | 4.50E-02 |
| VDR/RXR Activation | 1.19E+00 | 5.13E-02 |
| IL-12 Signaling and Production in Macrophages | 1.15E+00 | 4.39E-02 |
| Role of Pattern Recognition Receptors in Recognition of Bacteria and Viruses | 1.13E+00 | 4.31E-02 |
| Pentose Phosphate Pathway (Oxidative Branch) | 1.12E+00 | 2.50E-01 |
| Eumelanin Biosynthesis | 1.12E+00 | 2.50E-01 |
| IL-15 Production | 1.11E+00 | 8.33E-02 |
| D-myo-inositol (1,4,5,6)-Tetrakisphosphate Biosynthesis | 1.09E+00 | 4.20E-02 |
| D-myo-inositol (3,4,5,6)-tetrakisphosphate Biosynthesis | 1.09E+00 | 4.20E-02 |
| Acute Phase Response Signaling | 1.07E+00 | 3.82E-02 |
| Regulation of Cellular Mechanics by Calpain Protease | 1.06E+00 | 5.56E-02 |
| Apoptosis Signaling | 1.05E+00 | 4.60E-02 |
| LPS/IL-1 Mediated Inhibition of RXR Function | 1.05E+00 | 3.55E-02 |
| Serine Biosynthesis | 1.03E+00 | 2.00E-01 |
| Role of Hypercytokinemia/hyperchemokinemia in the Pathogenesis of Influenza | 1.02E+00 | 7.41E-02 |
| Sonic Hedgehog Signaling | 1.02E+00 | 7.41E-02 |
| Induction of Apoptosis by HIV1 | 9.75E-01 | 5.08E-02 |
| ATM Signaling | 9.75E-01 | 5.08E-02 |
| p53 Signaling | 9.61E-01 | 4.26E-02 |
| Glycerol Degradation I | 9.56E-01 | 1.67E-01 |
| Acetyl-CoA Biosynthesis I (Pyruvate Dehydrogenase Complex) | 9.56E-01 | 1.67E-01 |
| Human Embryonic Stem Cell Pluripotency | 9.46E-01 | 3.79E-02 |
| Mitotic Roles of Polo-Like Kinase | 9.42E-01 | 4.92E-02 |
| Amyotrophic Lateral Sclerosis Signaling | 9.37E-01 | 4.17E-02 |
| PCP pathway | 9.27E-01 | 4.84E-02 |
| Axonal Guidance Signaling | 9.23E-01 | 2.84E-02 |
| D-myo-inositol-5-phosphate Metabolism | 9.15E-01 | 3.70E-02 |
| 3-phosphoinositide Degradation | 9.06E-01 | 3.68E-02 |
| Triacylglycerol Biosynthesis | 8.97E-01 | 6.25E-02 |
| Superpathway of Serine and Glycine Biosynthesis I | 8.93E-01 | 1.43E-01 |
| TREM1 Signaling | 8.26E-01 | 4.35E-02 |
| Basal Cell Carcinoma Signaling | 8.26E-01 | 4.35E-02 |
| Thrombin Signaling | 8.24E-01 | 3.24E-02 |
| Role of Cytokines in Mediating Communication between Immune Cells | 8.14E-01 | 5.56E-02 |
| Androgen Signaling | 8.14E-01 | 3.74E-02 |
| 3-phosphoinositide Biosynthesis | 8.14E-01 | 3.42E-02 |
| Dendritic Cell Maturation | 8.05E-01 | 3.40E-02 |
| Role of NANOG in Mammalian Embryonic Stem Cell Pluripotency | 7.94E-01 | 3.67E-02 |
| p38 MAPK Signaling | 7.94E-01 | 3.67E-02 |
| Role of Lipids/Lipid Rafts in the Pathogenesis of Influenza | 7.92E-01 | 1.11E-01 |
| Protein Kinase A Signaling | 7.91E-01 | 2.78E-02 |
| Ephrin B Signaling | 7.88E-01 | 4.17E-02 |
| Granulocyte Adhesion and Diapedesis | 7.72E-01 | 3.31E-02 |
| Pentose Phosphate Pathway | 7.51E-01 | 1.00E-01 |
| Glucocorticoid Biosynthesis | 7.51E-01 | 1.00E-01 |
| PI3K/AKT Signaling | 7.09E-01 | 3.39E-02 |
| TR/RXR Activation | 6.85E-01 | 3.70E-02 |
| Assembly of RNA Polymerase III Complex | 6.80E-01 | 8.33E-02 |
| Retinoic acid Mediated Apoptosis Signaling | 6.80E-01 | 4.55E-02 |
| Role of Oct4 in Mammalian Embryonic Stem Cell Pluripotency | 6.65E-01 | 4.44E-02 |
| HIPPO signaling | 6.64E-01 | 3.61E-02 |
| Neuregulin Signaling | 6.54E-01 | 3.57E-02 |
| Hereditary Breast Cancer Signaling | 6.51E-01 | 3.20E-02 |
| Androgen Biosynthesis | 6.49E-01 | 7.69E-02 |
| Ubiquinol-10 Biosynthesis (Eukaryotic) | 6.49E-01 | 7.69E-02 |
| Guanosine Nucleotides Degradation III | 6.49E-01 | 7.69E-02 |
| TGF-β Signaling | 6.44E-01 | 3.53E-02 |
| G Beta Gamma Signaling | 6.34E-01 | 3.49E-02 |
| FAK Signaling | 6.34E-01 | 3.49E-02 |
| Salvage Pathways of Pyrimidine Ribonucleotides | 6.25E-01 | 3.45E-02 |
| Urate Biosynthesis/Inosine 5'-phosphate Degradation | 6.21E-01 | 7.14E-02 |
| Factors Promoting Cardiogenesis in Vertebrates | 6.15E-01 | 3.41E-02 |
| Nicotine Degradation II | 6.11E-01 | 4.08E-02 |
| Amyloid Processing | 6.11E-01 | 4.08E-02 |
| Cell Cycle: G2/M DNA Damage Checkpoint Regulation | 6.11E-01 | 4.08E-02 |
| IL-1 Signaling | 6.06E-01 | 3.37E-02 |
| Relaxin Signaling | 5.98E-01 | 3.03E-02 |
| Granzyme B Signaling | 5.95E-01 | 6.67E-02 |
| Chondroitin Sulfate Degradation (Metazoa) | 5.95E-01 | 6.67E-02 |
| Adenosine Nucleotides Degradation II | 5.95E-01 | 6.67E-02 |
| Hepatic Fibrosis / Hepatic Stellate Cell Activation | 5.93E-01 | 2.84E-02 |
| CD27 Signaling in Lymphocytes | 5.86E-01 | 3.92E-02 |
| eNOS Signaling | 5.83E-01 | 2.99E-02 |
| Methionine Degradation I (to Homocysteine) | 5.71E-01 | 6.25E-02 |
| Nitric Oxide Signaling in the Cardiovascular System | 5.63E-01 | 3.19E-02 |
| Superpathway of Inositol Phosphate Compounds | 5.52E-01 | 2.73E-02 |
| Mitochondrial L-carnitine Shuttle Pathway | 5.49E-01 | 5.88E-02 |
| Cholecystokinin/Gastrin-mediated Signaling | 5.38E-01 | 3.09E-02 |
| Oxidative Phosphorylation | 5.38E-01 | 3.09E-02 |
| Type I Diabetes Mellitus Signaling | 5.31E-01 | 3.06E-02 |
| Glutamate Receptor Signaling | 5.30E-01 | 3.57E-02 |
| Purine Nucleotides Degradation II (Aerobic) | 5.28E-01 | 5.56E-02 |
| Fatty Acid α-oxidation | 5.28E-01 | 5.56E-02 |
| Cysteine Biosynthesis III (mammalia) | 5.28E-01 | 5.56E-02 |
| Myc Mediated Apoptosis Signaling | 5.09E-01 | 3.45E-02 |
| DNA damage-induced 14-3-3σ Signaling | 5.08E-01 | 5.26E-02 |
| Rac Signaling | 5.00E-01 | 2.94E-02 |
| PXR/RXR Activation | 4.99E-01 | 3.39E-02 |
| Xenobiotic Metabolism Signaling | 4.99E-01 | 2.51E-02 |
| Endoplasmic Reticulum Stress Pathway | 4.90E-01 | 5.00E-02 |
| Eicosanoid Signaling | 4.80E-01 | 3.28E-02 |
| fMLP Signaling in Neutrophils | 4.79E-01 | 2.86E-02 |
| Hepatic Cholestasis | 4.75E-01 | 2.65E-02 |
| NAD Salvage Pathway II | 4.73E-01 | 4.76E-02 |
| Pyrimidine Deoxyribonucleotides De Novo Biosynthesis I | 4.73E-01 | 4.76E-02 |
| ERK5 Signaling | 4.71E-01 | 3.23E-02 |
| Corticotropin Releasing Hormone Signaling | 4.65E-01 | 2.80E-02 |
| T Helper Cell Differentiation | 4.62E-01 | 3.17E-02 |
| Role of Wnt/GSK-3β Signaling in the Pathogenesis of Influenza | 4.62E-01 | 3.17E-02 |
| Pyridoxal 5'-phosphate Salvage Pathway | 4.62E-01 | 3.17E-02 |
| Tumoricidal Function of Hepatic Natural Killer Cells | 4.57E-01 | 4.55E-02 |
| Bupropion Degradation | 4.57E-01 | 4.55E-02 |
| Dopamine-DARPP32 Feedback in cAMP Signaling | 4.53E-01 | 2.58E-02 |
| Mitochondrial Dysfunction | 4.48E-01 | 2.56E-02 |
| IL-10 Signaling | 4.45E-01 | 3.08E-02 |
| G-Protein Coupled Receptor Signaling | 4.42E-01 | 2.38E-02 |
| Glycolysis I | 4.41E-01 | 4.35E-02 |
| Acetone Degradation I (to Methylglyoxal) | 4.41E-01 | 4.35E-02 |
| Melatonin Signaling | 4.36E-01 | 3.03E-02 |
| IL-6 Signaling | 4.27E-01 | 2.65E-02 |
| Cytotoxic T Lymphocyte-mediated Apoptosis of Target Cells | 4.27E-01 | 4.17E-02 |
| Estrogen-mediated S-phase Entry | 4.27E-01 | 4.17E-02 |
| Gluconeogenesis I | 4.27E-01 | 4.17E-02 |
| NF-κB Signaling | 4.22E-01 | 2.48E-02 |
| cAMP-mediated signaling | 4.08E-01 | 2.36E-02 |
| Endothelin-1 Signaling | 4.07E-01 | 2.44E-02 |
| Role of Osteoblasts, Osteoclasts and Chondrocytes in Rheumatoid Arthritis | 4.03E-01 | 2.35E-02 |
| LPS-stimulated MAPK Signaling | 3.97E-01 | 2.82E-02 |
| BMP signaling pathway | 3.90E-01 | 2.78E-02 |
| NRF2-mediated Oxidative Stress Response | 3.88E-01 | 2.38E-02 |
| Production of Nitric Oxide and Reactive Oxygen Species in Macrophages | 3.88E-01 | 2.38E-02 |
| Pyrimidine Ribonucleotides Interconversion | 3.87E-01 | 3.70E-02 |
| Antigen Presentation Pathway | 3.87E-01 | 3.70E-02 |
| Huntington's Disease Signaling | 3.87E-01 | 2.30E-02 |
| Ephrin Receptor Signaling | 3.74E-01 | 2.34E-02 |
| Pyrimidine Ribonucleotides De Novo Biosynthesis | 3.64E-01 | 3.45E-02 |
| AMPK Signaling | 3.61E-01 | 2.30E-02 |
| Role of RIG1-like Receptors in Antiviral Innate Immunity | 3.53E-01 | 3.33E-02 |
| ERK/MAPK Signaling | 3.53E-01 | 2.27E-02 |
| Ceramide Signaling | 3.50E-01 | 2.56E-02 |
| Signaling by Rho Family GTPases | 3.48E-01 | 2.20E-02 |
| Superpathway of Methionine Degradation | 3.42E-01 | 3.23E-02 |
| Prostate Cancer Signaling | 3.37E-01 | 2.50E-02 |
| Aryl Hydrocarbon Receptor Signaling | 3.36E-01 | 2.31E-02 |
| Circadian Rhythm Signaling | 3.32E-01 | 3.12E-02 |
| GPCR-Mediated Nutrient Sensing in Enteroendocrine Cells | 3.31E-01 | 2.47E-02 |
| Breast Cancer Regulation by Stathmin1 | 3.24E-01 | 2.19E-02 |
| Oncostatin M Signaling | 3.23E-01 | 3.03E-02 |
| Complement System | 3.23E-01 | 3.03E-02 |
| TWEAK Signaling | 3.14E-01 | 2.94E-02 |
| tRNA Splicing | 3.14E-01 | 2.94E-02 |
| Estrogen Biosynthesis | 3.14E-01 | 2.94E-02 |
| Synaptic Long Term Depression | 3.09E-01 | 2.21E-02 |
| Primary Immunodeficiency Signaling | 3.05E-01 | 2.86E-02 |
| Graft-versus-Host Disease Signaling | 3.05E-01 | 2.86E-02 |
| SAPK/JNK Signaling | 2.97E-01 | 2.30E-02 |
| Notch Signaling | 2.96E-01 | 2.78E-02 |
| Protein Ubiquitination Pathway | 2.94E-01 | 2.06E-02 |
| Inhibition of Matrix Metalloproteases | 2.88E-01 | 2.70E-02 |
| Molecular Mechanisms of Cancer | 2.76E-01 | 1.98E-02 |
| Role of PKR in Interferon Induction and Antiviral Response | 2.73E-01 | 2.56E-02 |
| Mechanisms of Viral Exit from Host Cells | 2.73E-01 | 2.56E-02 |
| Docosahexaenoic Acid (DHA) Signaling | 2.73E-01 | 2.56E-02 |
| Thyroid Cancer Signaling | 2.73E-01 | 2.56E-02 |
| Integrin Signaling | 2.71E-01 | 2.02E-02 |
| Telomerase Signaling | 2.68E-01 | 2.15E-02 |
| Aldosterone Signaling in Epithelial Cells | 2.65E-01 | 2.04E-02 |
| Mouse Embryonic Stem Cell Pluripotency | 2.63E-01 | 2.13E-02 |
| IGF-1 Signaling | 2.63E-01 | 2.13E-02 |
| Tec Kinase Signaling | 2.61E-01 | 2.03E-02 |
| Nicotine Degradation III | 2.58E-01 | 2.44E-02 |
| Melatonin Degradation I | 2.58E-01 | 2.44E-02 |
| CXCR4 Signaling | 2.57E-01 | 2.01E-02 |
| UVC-Induced MAPK Signaling | 2.52E-01 | 2.38E-02 |
| Gap Junction Signaling | 2.50E-01 | 1.99E-02 |
| HGF Signaling | 2.29E-01 | 1.96E-02 |
| Superpathway of Melatonin Degradation | 2.27E-01 | 2.17E-02 |
| Nur77 Signaling in T Lymphocytes | 2.21E-01 | 2.13E-02 |
| TNFR1 Signaling | 2.21E-01 | 2.13E-02 |
| Phototransduction Pathway | 2.15E-01 | 2.08E-02 |
| Activation of IRF by Cytosolic Pattern Recognition Receptors | 2.05E-01 | 2.00E-02 |
| Role of PI3K/AKT Signaling in the Pathogenesis of Influenza | 2.05E-01 | 2.00E-02 |
| UVB-Induced MAPK Signaling | 2.05E-01 | 2.00E-02 |
| Semaphorin Signaling in Neurons | 2.00E-01 | 1.96E-02 |

**Table S4 Canonical pathways of SMI VS eNOS KO.**

| **Ingenuity Canonical Pathways** | **-log(p-value)** | **Ratio** |
| --- | --- | --- |
| Bladder Cancer Signaling | 2.86E+00 | 9.52E-02 |
| Dendritic Cell Maturation | 2.84E+00 | 7.48E-02 |
| Cell Cycle: G2/M DNA Damage Checkpoint Regulation | 2.82E+00 | 1.22E-01 |
| Hepatic Fibrosis / Hepatic Stellate Cell Activation | 2.71E+00 | 6.82E-02 |
| HIF1α Signaling | 2.46E+00 | 8.25E-02 |
| ATM Signaling | 2.41E+00 | 1.02E-01 |
| Hypoxia Signaling in the Cardiovascular System | 2.27E+00 | 9.52E-02 |
| Systemic Lupus Erythematosus Signaling | 2.12E+00 | 6.33E-02 |
| GADD45 Signaling | 1.92E+00 | 1.58E-01 |
| G-Protein Coupled Receptor Signaling | 1.88E+00 | 5.16E-02 |
| Prostate Cancer Signaling | 1.78E+00 | 7.50E-02 |
| Induction of Apoptosis by HIV1 | 1.76E+00 | 8.47E-02 |
| FGF Signaling | 1.66E+00 | 7.06E-02 |
| CD40 Signaling | 1.65E+00 | 7.94E-02 |
| Communication between Innate and Adaptive Immune Cells | 1.65E+00 | 7.94E-02 |
| Estrogen-mediated S-phase Entry | 1.65E+00 | 1.25E-01 |
| NF-κB Signaling | 1.64E+00 | 5.59E-02 |
| Huntington's Disease Signaling | 1.61E+00 | 5.07E-02 |
| L-dopachrome Biosynthesis | 1.59E+00 | 1.00E+00 |
| IL-6 Signaling | 1.58E+00 | 6.19E-02 |
| IL-1 Signaling | 1.57E+00 | 6.74E-02 |
| IL-12 Signaling and Production in Macrophages | 1.56E+00 | 6.14E-02 |
| Role of Pattern Recognition Receptors in Recognition of Bacteria and Viruses | 1.53E+00 | 6.03E-02 |
| Intrinsic Prothrombin Activation Pathway | 1.51E+00 | 1.11E-01 |
| p53 Signaling | 1.47E+00 | 6.38E-02 |
| Nitric Oxide Signaling in the Cardiovascular System | 1.47E+00 | 6.38E-02 |
| Small Cell Lung Cancer Signaling | 1.45E+00 | 7.04E-02 |
| Amyotrophic Lateral Sclerosis Signaling | 1.44E+00 | 6.25E-02 |
| Activation of IRF by Cytosolic Pattern Recognition Receptors | 1.41E+00 | 8.00E-02 |
| Molecular Mechanisms of Cancer | 1.40E+00 | 4.24E-02 |
| Regulation of the Epithelial-Mesenchymal Transition Pathway | 1.40E+00 | 5.06E-02 |
| CD27 Signaling in Lymphocytes | 1.39E+00 | 7.84E-02 |
| Pancreatic Adenocarcinoma Signaling | 1.29E+00 | 5.77E-02 |
| Inhibition of Angiogenesis by TSP1 | 1.29E+00 | 9.09E-02 |
| Complement System | 1.29E+00 | 9.09E-02 |
| Relaxin Signaling | 1.27E+00 | 5.30E-02 |
| Chondroitin Sulfate Degradation (Metazoa) | 1.26E+00 | 1.33E-01 |
| Primary Immunodeficiency Signaling | 1.22E+00 | 8.57E-02 |
| Myc Mediated Apoptosis Signaling | 1.21E+00 | 6.90E-02 |
| p38 MAPK Signaling | 1.21E+00 | 5.50E-02 |
| Inhibition of Matrix Metalloproteases | 1.17E+00 | 8.11E-02 |
| Mitotic Roles of Polo-Like Kinase | 1.15E+00 | 6.56E-02 |
| Apoptosis Signaling | 1.14E+00 | 5.75E-02 |
| Clathrin-mediated Endocytosis Signaling | 1.11E+00 | 4.62E-02 |
| T Helper Cell Differentiation | 1.11E+00 | 6.35E-02 |
| Protein Kinase A Signaling | 1.09E+00 | 3.89E-02 |
| Actin Cytoskeleton Signaling | 1.09E+00 | 4.39E-02 |
| B Cell Activating Factor Signaling | 1.09E+00 | 7.50E-02 |
| PI3K/AKT Signaling | 1.08E+00 | 5.08E-02 |
| Granulocyte Adhesion and Diapedesis | 1.03E+00 | 4.64E-02 |
| cAMP-mediated signaling | 1.02E+00 | 4.25E-02 |
| Pentose Phosphate Pathway (Oxidative Branch) | 1.01E+00 | 2.50E-01 |
| Eumelanin Biosynthesis | 1.01E+00 | 2.50E-01 |
| Myo-inositol Biosynthesis | 1.01E+00 | 2.50E-01 |
| Melatonin Degradation II | 1.01E+00 | 2.50E-01 |
| Cdc42 Signaling | 1.00E+00 | 4.84E-02 |
| TREM1 Signaling | 9.98E-01 | 5.80E-02 |
| Basal Cell Carcinoma Signaling | 9.98E-01 | 5.80E-02 |
| Oxidative Phosphorylation | 9.85E-01 | 5.15E-02 |
| Type I Diabetes Mellitus Signaling | 9.71E-01 | 5.10E-02 |
| LPS-stimulated MAPK Signaling | 9.64E-01 | 5.63E-02 |
| Role of Macrophages, Fibroblasts and Endothelial Cells in Rheumatoid Arthritis | 9.58E-01 | 3.90E-02 |
| B Cell Development | 9.34E-01 | 8.70E-02 |
| Agranulocyte Adhesion and Diapedesis | 9.21E-01 | 4.35E-02 |
| Creatine-phosphate Biosynthesis | 9.15E-01 | 2.00E-01 |
| Serine Biosynthesis | 9.15E-01 | 2.00E-01 |
| Human Embryonic Stem Cell Pluripotency | 9.09E-01 | 4.55E-02 |
| Cytotoxic T Lymphocyte-mediated Apoptosis of Target Cells | 9.04E-01 | 8.33E-02 |
| Gluconeogenesis I | 9.04E-01 | 8.33E-02 |
| Cyclins and Cell Cycle Regulation | 9.00E-01 | 5.33E-02 |
| Endothelin-1 Signaling | 8.90E-01 | 4.27E-02 |
| B Cell Receptor Signaling | 8.90E-01 | 4.27E-02 |
| eNOS Signaling | 8.86E-01 | 4.48E-02 |
| Altered T Cell and B Cell Signaling in Rheumatoid Arthritis | 8.70E-01 | 5.19E-02 |
| Role of PI3K/AKT Signaling in the Pathogenesis of Influenza | 8.67E-01 | 6.00E-02 |
| Role of IL-17A in Arthritis | 8.67E-01 | 6.00E-02 |
| Renin-Angiotensin Signaling | 8.54E-01 | 4.67E-02 |
| Corticotropin Releasing Hormone Signaling | 8.54E-01 | 4.67E-02 |
| Glycerol Degradation I | 8.41E-01 | 1.67E-01 |
| Acetyl-CoA Biosynthesis I (Pyruvate Dehydrogenase Complex) | 8.41E-01 | 1.67E-01 |
| Salvage Pathways of Pyrimidine Deoxyribonucleotides | 8.41E-01 | 1.67E-01 |
| Role of Hypercytokinemia/hyperchemokinemia in the Pathogenesis of Influenza | 8.20E-01 | 7.41E-02 |
| Sonic Hedgehog Signaling | 8.20E-01 | 7.41E-02 |
| OX40 Signaling Pathway | 8.13E-01 | 5.66E-02 |
| Atherosclerosis Signaling | 8.07E-01 | 4.50E-02 |
| ILK Signaling | 7.97E-01 | 4.02E-02 |
| Regulation of Cellular Mechanics by Calpain Protease | 7.96E-01 | 5.56E-02 |
| Melanocyte Development and Pigmentation Signaling | 7.87E-01 | 4.82E-02 |
| HMGB1 Signaling | 7.85E-01 | 4.42E-02 |
| Airway Pathology in Chronic Obstructive Pulmonary Disease | 7.80E-01 | 1.43E-01 |
| Superpathway of Serine and Glycine Biosynthesis I | 7.80E-01 | 1.43E-01 |
| IL-8 Signaling | 7.71E-01 | 3.95E-02 |
| 3-phosphoinositide Biosynthesis | 7.64E-01 | 4.11E-02 |
| FAK Signaling | 7.48E-01 | 4.65E-02 |
| RANK Signaling in Osteoclasts | 7.48E-01 | 4.65E-02 |
| Role of RIG1-like Receptors in Antiviral Innate Immunity | 7.48E-01 | 6.67E-02 |
| 4-1BB Signaling in T Lymphocytes | 7.48E-01 | 6.67E-02 |
| Role of Osteoblasts, Osteoclasts and Chondrocytes in Rheumatoid Arthritis | 7.42E-01 | 3.76E-02 |
| CXCR4 Signaling | 7.36E-01 | 4.03E-02 |
| SAPK/JNK Signaling | 7.36E-01 | 4.60E-02 |
| Gαi Signaling | 7.32E-01 | 4.24E-02 |
| Sphingomyelin Metabolism | 7.27E-01 | 1.25E-01 |
| Autoimmune Thyroid Disease Signaling | 7.26E-01 | 6.45E-02 |
| MIF-mediated Glucocorticoid Regulation | 7.26E-01 | 6.45E-02 |
| D-myo-inositol (1,4,5,6)-Tetrakisphosphate Biosynthesis | 7.21E-01 | 4.20E-02 |
| D-myo-inositol (3,4,5,6)-tetrakisphosphate Biosynthesis | 7.21E-01 | 4.20E-02 |
| Hepatic Cholestasis | 7.18E-01 | 3.97E-02 |
| PPAR Signaling | 7.12E-01 | 4.49E-02 |
| Circadian Rhythm Signaling | 7.05E-01 | 6.25E-02 |
| IL-17A Signaling in Airway Cells | 7.02E-01 | 5.00E-02 |
| Cell Cycle: G1/S Checkpoint Regulation | 6.88E-01 | 4.92E-02 |
| Role of Lipids/Lipid Rafts in the Pathogenesis of Influenza | 6.81E-01 | 1.11E-01 |
| Mitochondrial Dysfunction | 6.76E-01 | 3.85E-02 |
| ERK5 Signaling | 6.74E-01 | 4.84E-02 |
| GNRH Signaling | 6.73E-01 | 4.03E-02 |
| TWEAK Signaling | 6.66E-01 | 5.88E-02 |
| Hereditary Breast Cancer Signaling | 6.64E-01 | 4.00E-02 |
| Role of Wnt/GSK-3β Signaling in the Pathogenesis of Influenza | 6.61E-01 | 4.76E-02 |
| Glucocorticoid Receptor Signaling | 6.50E-01 | 3.46E-02 |
| Embryonic Stem Cell Differentiation into Cardiac Lineages | 6.41E-01 | 1.00E-01 |
| Pentose Phosphate Pathway | 6.41E-01 | 1.00E-01 |
| Glucocorticoid Biosynthesis | 6.41E-01 | 1.00E-01 |
| Angiopoietin Signaling | 6.35E-01 | 4.62E-02 |
| Role of Cytokines in Mediating Communication between Immune Cells | 6.29E-01 | 5.56E-02 |
| Paxillin Signaling | 6.25E-01 | 4.12E-02 |
| Neurotrophin/TRK Signaling | 6.22E-01 | 4.55E-02 |
| Aryl Hydrocarbon Receptor Signaling | 6.19E-01 | 3.85E-02 |
| April Mediated Signaling | 6.12E-01 | 5.41E-02 |
| GDNF Family Ligand-Receptor Interactions | 6.10E-01 | 4.48E-02 |
| GPCR-Mediated Integration of Enteroendocrine Signaling Exemplified by an L Cell | 6.10E-01 | 4.48E-02 |
| iCOS-iCOSL Signaling in T Helper Cells | 6.05E-01 | 4.04E-02 |
| Calcium Signaling | 5.97E-01 | 3.61E-02 |
| Transcriptional Regulatory Network in Embryonic Stem Cells | 5.95E-01 | 5.26E-02 |
| CREB Signaling in Neurons | 5.90E-01 | 3.59E-02 |
| Caveolar-mediated Endocytosis Signaling | 5.86E-01 | 4.35E-02 |
| Production of Nitric Oxide and Reactive Oxygen Species in Macrophages | 5.83E-01 | 3.57E-02 |
| Role of PKR in Interferon Induction and Antiviral Response | 5.79E-01 | 5.13E-02 |
| MIF Regulation of Innate Immunity | 5.79E-01 | 5.13E-02 |
| Docosahexaenoic Acid (DHA) Signaling | 5.79E-01 | 5.13E-02 |
| D-myo-inositol-5-phosphate Metabolism | 5.78E-01 | 3.70E-02 |
| HGF Signaling | 5.77E-01 | 3.92E-02 |
| Toll-like Receptor Signaling | 5.74E-01 | 4.29E-02 |
| Assembly of RNA Polymerase III Complex | 5.73E-01 | 8.33E-02 |
| 3-phosphoinositide Degradation | 5.70E-01 | 3.68E-02 |
| Sphingosine-1-phosphate Signaling | 5.67E-01 | 3.88E-02 |
| Serotonin Receptor Signaling | 5.64E-01 | 5.00E-02 |
| Leptin Signaling in Obesity | 5.52E-01 | 4.17E-02 |
| Ephrin B Signaling | 5.52E-01 | 4.17E-02 |
| fMLP Signaling in Neutrophils | 5.50E-01 | 3.81E-02 |
| NGF Signaling | 5.50E-01 | 3.81E-02 |
| FcγRIIB Signaling in B Lymphocytes | 5.49E-01 | 4.88E-02 |
| Androgen Biosynthesis | 5.43E-01 | 7.69E-02 |
| Ubiquinol-10 Biosynthesis (Eukaryotic) | 5.43E-01 | 7.69E-02 |
| Guanosine Nucleotides Degradation III | 5.43E-01 | 7.69E-02 |
| STAT3 Pathway | 5.42E-01 | 4.11E-02 |
| Melanoma Signaling | 5.34E-01 | 4.76E-02 |
| HER-2 Signaling in Breast Cancer | 5.31E-01 | 4.05E-02 |
| VEGF Family Ligand-Receptor Interactions | 5.31E-01 | 4.05E-02 |
| ERK/MAPK Signaling | 5.28E-01 | 3.41E-02 |
| CD28 Signaling in T Helper Cells | 5.24E-01 | 3.70E-02 |
| iNOS Signaling | 5.20E-01 | 4.65E-02 |
| Urate Biosynthesis/Inosine 5'-phosphate Degradation | 5.16E-01 | 7.14E-02 |
| Phenylalanine Degradation IV (Mammalian, via Side Chain) | 5.16E-01 | 7.14E-02 |
| PKCθ Signaling in T Lymphocytes | 5.15E-01 | 3.67E-02 |
| Retinoic acid Mediated Apoptosis Signaling | 5.07E-01 | 4.55E-02 |
| MSP-RON Signaling Pathway | 5.07E-01 | 4.55E-02 |
| Cardiac Hypertrophy Signaling | 5.04E-01 | 3.26E-02 |
| Regulation of IL-2 Expression in Activated and Anergic T Lymphocytes | 5.01E-01 | 3.90E-02 |
| Role of Oct4 in Mammalian Embryonic Stem Cell Pluripotency | 4.94E-01 | 4.44E-02 |
| nNOS Signaling in Neurons | 4.94E-01 | 4.44E-02 |
| Granzyme B Signaling | 4.92E-01 | 6.67E-02 |
| Adenosine Nucleotides Degradation II | 4.92E-01 | 6.67E-02 |
| Ceramide Signaling | 4.92E-01 | 3.85E-02 |
| Superpathway of Inositol Phosphate Compounds | 4.84E-01 | 3.28E-02 |
| Tec Kinase Signaling | 4.82E-01 | 3.38E-02 |
| Reelin Signaling in Neurons | 4.82E-01 | 3.80E-02 |
| Superpathway of Melatonin Degradation | 4.81E-01 | 4.35E-02 |
| Type II Diabetes Mellitus Signaling | 4.76E-01 | 3.51E-02 |
| Gα12/13 Signaling | 4.76E-01 | 3.51E-02 |
| Thrombin Signaling | 4.72E-01 | 3.24E-02 |
| Nur77 Signaling in T Lymphocytes | 4.69E-01 | 4.26E-02 |
| TNFR1 Signaling | 4.69E-01 | 4.26E-02 |
| Dermatan Sulfate Degradation (Metazoa) | 4.69E-01 | 6.25E-02 |
| TR/RXR Activation | 4.64E-01 | 3.70E-02 |
| Phototransduction Pathway | 4.57E-01 | 4.17E-02 |
| Cardiomyocyte Differentiation via BMP Receptors | 4.48E-01 | 5.88E-02 |
| Lipid Antigen Presentation by CD1 | 4.48E-01 | 5.88E-02 |
| HIPPO signaling | 4.47E-01 | 3.61E-02 |
| Amyloid Processing | 4.46E-01 | 4.08E-02 |
| Signaling by Rho Family GTPases | 4.40E-01 | 3.08E-02 |
| UVB-Induced MAPK Signaling | 4.35E-01 | 4.00E-02 |
| PAK Signaling | 4.30E-01 | 3.53E-02 |
| Purine Nucleotides Degradation II (Aerobic) | 4.28E-01 | 5.56E-02 |
| Fatty Acid α-oxidation | 4.28E-01 | 5.56E-02 |
| Acute Phase Response Signaling | 4.26E-01 | 3.18E-02 |
| Cellular Effects of Sildenafil (Viagra) | 4.26E-01 | 3.31E-02 |
| G Beta Gamma Signaling | 4.22E-01 | 3.49E-02 |
| Colorectal Cancer Metastasis Signaling | 4.21E-01 | 3.03E-02 |
| Role of NFAT in Regulation of the Immune Response | 4.14E-01 | 3.14E-02 |
| Salvage Pathways of Pyrimidine Ribonucleotides | 4.14E-01 | 3.45E-02 |
| Endometrial Cancer Signaling | 4.14E-01 | 3.85E-02 |
| Putrescine Degradation III | 4.10E-01 | 5.26E-02 |
| DNA damage-induced 14-3-3σ Signaling | 4.10E-01 | 5.26E-02 |
| VEGF Signaling | 4.06E-01 | 3.41E-02 |
| Lymphotoxin β Receptor Signaling | 4.03E-01 | 3.77E-02 |
| Role of CHK Proteins in Cell Cycle Checkpoint Control | 4.03E-01 | 3.77E-02 |
| Integrin Signaling | 4.02E-01 | 3.03E-02 |
| Axonal Guidance Signaling | 4.02E-01 | 2.84E-02 |
| Tryptophan Degradation X (Mammalian, via Tryptamine) | 3.93E-01 | 5.00E-02 |
| Wnt/Ca+ pathway | 3.84E-01 | 3.64E-02 |
| NAD Salvage Pathway II | 3.77E-01 | 4.76E-02 |
| Pyrimidine Deoxyribonucleotides De Novo Biosynthesis I | 3.77E-01 | 4.76E-02 |
| Ovarian Cancer Signaling | 3.75E-01 | 3.10E-02 |
| Glioma Invasiveness Signaling | 3.75E-01 | 3.57E-02 |
| Glutamate Receptor Signaling | 3.75E-01 | 3.57E-02 |
| Telomerase Signaling | 3.70E-01 | 3.23E-02 |
| Antioxidant Action of Vitamin C | 3.70E-01 | 3.23E-02 |
| Mouse Embryonic Stem Cell Pluripotency | 3.63E-01 | 3.19E-02 |
| IGF-1 Signaling | 3.63E-01 | 3.19E-02 |
| Tumoricidal Function of Hepatic Natural Killer Cells | 3.62E-01 | 4.55E-02 |
| IL-17A Signaling in Gastric Cells | 3.62E-01 | 4.55E-02 |
| Bupropion Degradation | 3.62E-01 | 4.55E-02 |
| Ephrin Receptor Signaling | 3.51E-01 | 2.92E-02 |
| Dopamine Degradation | 3.47E-01 | 4.35E-02 |
| Glycolysis I | 3.47E-01 | 4.35E-02 |
| Acetone Degradation I (to Methylglyoxal) | 3.47E-01 | 4.35E-02 |
| Cholecystokinin/Gastrin-mediated Signaling | 3.43E-01 | 3.09E-02 |
| Glioma Signaling | 3.43E-01 | 3.09E-02 |
| IL-22 Signaling | 3.34E-01 | 4.17E-02 |
| IL-15 Production | 3.34E-01 | 4.17E-02 |
| Role of JAK1 and JAK3 in γc Cytokine Signaling | 3.32E-01 | 3.28E-02 |
| CCR5 Signaling in Macrophages | 3.24E-01 | 3.23E-02 |
| Estrogen-Dependent Breast Cancer Signaling | 3.24E-01 | 3.23E-02 |
| PCP pathway | 3.24E-01 | 3.23E-02 |
| Role of JAK family kinases in IL-6-type Cytokine Signaling | 3.21E-01 | 4.00E-02 |
| Epithelial Adherens Junction Signaling | 3.21E-01 | 2.88E-02 |
| Antiproliferative Role of Somatostatin Receptor 2 | 3.17E-01 | 3.17E-02 |
| Pyridoxal 5'-phosphate Salvage Pathway | 3.17E-01 | 3.17E-02 |
| Remodeling of Epithelial Adherens Junctions | 3.17E-01 | 3.17E-02 |
| Rac Signaling | 3.13E-01 | 2.94E-02 |
| Antiproliferative Role of TOB in T Cell Signaling | 3.09E-01 | 3.85E-02 |
| IL-10 Signaling | 3.02E-01 | 3.08E-02 |
| Role of Tissue Factor in Cancer | 3.01E-01 | 2.88E-02 |
| Pyrimidine Ribonucleotides Interconversion | 2.98E-01 | 3.70E-02 |
| Antigen Presentation Pathway | 2.98E-01 | 3.70E-02 |
| IL-17 Signaling | 2.95E-01 | 3.03E-02 |
| Agrin Interactions at Neuromuscular Junction | 2.95E-01 | 3.03E-02 |
| Renal Cell Carcinoma Signaling | 2.95E-01 | 3.03E-02 |
| Erythropoietin Signaling | 2.88E-01 | 2.99E-02 |
| Hematopoiesis from Pluripotent Stem Cells | 2.87E-01 | 3.57E-02 |
| Role of p14/p19ARF in Tumor Suppression | 2.87E-01 | 3.57E-02 |
| Androgen Signaling | 2.85E-01 | 2.80E-02 |
| LXR/RXR Activation | 2.80E-01 | 2.78E-02 |
| Gustation Pathway | 2.80E-01 | 2.78E-02 |
| TNFR2 Signaling | 2.77E-01 | 3.45E-02 |
| Pyrimidine Ribonucleotides De Novo Biosynthesis | 2.77E-01 | 3.45E-02 |
| Crosstalk between Dendritic Cells and Natural Killer Cells | 2.75E-01 | 2.90E-02 |
| Role of NANOG in Mammalian Embryonic Stem Cell Pluripotency | 2.75E-01 | 2.75E-02 |
| Leukocyte Extravasation Signaling | 2.69E-01 | 2.63E-02 |
| PEDF Signaling | 2.69E-01 | 2.86E-02 |
| IL-4 Signaling | 2.69E-01 | 2.86E-02 |
| Gap Junction Signaling | 2.65E-01 | 2.65E-02 |
| FLT3 Signaling in Hematopoietic Progenitor Cells | 2.63E-01 | 2.82E-02 |
| Prolactin Signaling | 2.57E-01 | 2.78E-02 |
| JAK/Stat Signaling | 2.57E-01 | 2.78E-02 |
| BMP signaling pathway | 2.57E-01 | 2.78E-02 |
| NF-κB Activation by Viruses | 2.51E-01 | 2.74E-02 |
| 14-3-3-mediated Signaling | 2.50E-01 | 2.63E-02 |
| Dopamine-DARPP32 Feedback in cAMP Signaling | 2.49E-01 | 2.58E-02 |
| IL-17A Signaling in Fibroblasts | 2.49E-01 | 3.12E-02 |
| Noradrenaline and Adrenaline Degradation | 2.49E-01 | 3.12E-02 |
| Dopamine Receptor Signaling | 2.45E-01 | 2.70E-02 |
| P2Y Purigenic Receptor Signaling Pathway | 2.41E-01 | 2.59E-02 |
| IL-9 Signaling | 2.32E-01 | 2.94E-02 |
| tRNA Splicing | 2.32E-01 | 2.94E-02 |
| Estrogen Biosynthesis | 2.32E-01 | 2.94E-02 |
| CTLA4 Signaling in Cytotoxic T Lymphocytes | 2.29E-01 | 2.60E-02 |
| PDGF Signaling | 2.29E-01 | 2.60E-02 |
| Graft-versus-Host Disease Signaling | 2.24E-01 | 2.86E-02 |
| Neuroprotective Role of THOP1 in Alzheimer's Disease | 2.24E-01 | 2.86E-02 |
| Role of BRCA1 in DNA Damage Response | 2.24E-01 | 2.56E-02 |
| Acute Myeloid Leukemia Signaling | 2.24E-01 | 2.56E-02 |
| Notch Signaling | 2.17E-01 | 2.78E-02 |
| autophagy | 2.17E-01 | 2.78E-02 |
| Role of IL-17F in Allergic Inflammatory Airway Diseases | 2.09E-01 | 2.70E-02 |
| Thyroid Cancer Signaling | 1.96E-01 | 2.56E-02 |

**Table S5 Disease and function of SFI VS eNOS KO.**

| **Categories** | **Diseases or Functions Annotation** | **p-Value** | **Molecules** |
| --- | --- | --- | --- |
| Congenital Heart Anomaly | familial cardiac septal defect | 7.72E-05 | CITED2,DVL3,GATA4,GATA6,LEFTY1,PBX1,ZFPM2 |
| Cardiac Hypertrophy | ventricular hypertrophy | 2.49E-04 | CPT1A,FKBP1A,FKBP1B,GATA4,IL18,INHBA,NT5E,PBX1,PDE5A,VDR |
| Congenital Heart Anomaly | familial atrial septal defect | 3.74E-04 | CITED2,GATA4,GATA6 |
| Congenital Heart Anomaly | persistent truncus arteriosus | 4.61E-04 | CITED2,DVL3,GATA4,GATA6,PBX1 |
| Congenital Heart Anomaly | familial ventricular septal defect | 4.82E-04 | CITED2,DVL3,GATA4,LEFTY1,PBX1,ZFPM2 |
| Congenital Heart Anomaly | tetralogy of Fallot | 5.53E-04 | GATA4,GATA6,ZFPM2 |
| Congenital Heart Anomaly | conotruncal heart malformations | 7.18E-04 | CITED2,DVL3,GATA4,GATA6,LEFTY1,PBX1,ZFPM2 |
| Congenital Heart Anomaly | atrioventricular septal defect and common atrioventricular junction | 7.64E-04 | GATA4,GATA6,LEFTY1,ZFPM2 |
| Congenital Heart Anomaly | hypoplasia of aortic arch | 1.11E-03 | GATA4,GATA6 |
| Congenital Heart Anomaly | ventricular septal defect | 1.19E-03 | CITED2,DVL3,FKBP1A,GATA4,GATA6,LEFTY1,PBX1,ZFPM2 |
| Congenital Heart Anomaly | atrial septal defect | 1.70E-03 | CITED2,GATA4,GATA6,LEFTY1,ZFPM2 |
| Congenital Heart Anomaly | atresia of tricuspid valve | 2.18E-03 | LEFTY1,ZFPM2 |
| Congenital Heart Anomaly | double outlet right ventricle | 2.50E-03 | CITED2,DVL3,LEFTY1,PBX1,ZFPM2 |
| Cardiac Stenosis | stenosis of pulmonary artery | 3.59E-03 | CITED2,IGFBP7 |
| Cardiac Hypertrophy | hypertrophy of left ventricle | 5.43E-03 | CPT1A,FKBP1A,FKBP1B,NT5E,PBX1,VDR |
| Cardiac Hypertrophy | hypertrophy of ventricular myocytes | 1.07E-02 | GATA4,IL18,INHBA |
| Congenital Heart Anomaly | atrial septal defect type 2 | 1.93E-02 | GATA4 |
| Congenital Heart Anomaly | atrial septal defect type 8 | 1.93E-02 | CITED2 |
| Congenital Heart Anomaly | atrial septal defect type 9 | 1.93E-02 | GATA6 |
| Congenital Heart Anomaly | atrioventricular septal defect type 4 | 1.93E-02 | GATA4 |
| Congenital Heart Anomaly | atrioventricular septal defect type 5 | 1.93E-02 | GATA6 |
| Cardiac Inflammation | chronic inflammation of heart | 1.93E-02 | IFNA4 |
| Glomerular Injury | delayed hypersensitive reaction of renal glomerulus | 1.93E-02 | IL18 |
| Glomerular Injury | focal segmental glomerulosclerosis type 6 | 1.93E-02 | MYO1E |
| Cardiac Hypoplasia | hypoplasia of ventricular compact zone | 1.93E-02 | ZFPM2 |
| Renal Damage, Renal Tubule Injury | injury of renal tubular epithelial cells | 1.93E-02 | RGMB |
| Congenital Heart Anomaly | pancreatic agenesis and congenital heart defects | 1.93E-02 | GATA6 |
| Cardiac Stenosis | retinal arterial macroaneurysm with supravalvular pulmonic stenosis | 1.93E-02 | IGFBP7 |
| Liver Cirrhosis | stage 3 primary biliary cirrhosis | 1.93E-02 | COL3A1 |
| Liver Cirrhosis | stage 4 primary biliary cirrhosis | 1.93E-02 | COL3A1 |
| Congenital Heart Anomaly | ventricular septal defect type 1 | 1.93E-02 | GATA4 |
| Congenital Heart Anomaly | ventricular septal defect type 2 | 1.93E-02 | CITED2 |
| Cardiac Dysfunction | systolic dysfunction | 1.98E-02 | FKBP1A,MARCKSL1,PDE5A |
| Cardiac Hypoplasia | hypoplasia of myocardium | 2.53E-02 | MYCN,ZFPM2 |
| Increased Levels of AST | increased localization of AST | 3.32E-02 | MAP3K8,SAA1 |
| Kidney Failure | chronic renal failure | 3.42E-02 | AMY2B,BUB1B,DCN,FKBP1A,MYO1E,PDE5A,VDR |
| Liver Damage | damage of liver | 3.47E-02 | AMY2B,COL3A1,FKBP1A,IL18,INHBA,NT5E,PAWR,PRKCZ,SAA1,SERPING1 |
| Cardiac Arrythmia | cardiac fibrillation | 3.70E-02 | AMY2B,CHI3L1,COL3A1,FKBP1B,NHLH1,TUBD1 |
| Cardiac Hypertrophy | hypertrophy of heart | 3.75E-02 | CPT1A,FKBP1A,FKBP1B,GATA4,GATA6,IL18,INHBA,LEPR,NT5E,PBX1,PDE5A,TERT,VDR |
| Liver Cholestasis | accumulation of taurocholic acid | 3.83E-02 | VDR |
| Cardiac Inflammation | acute myocarditis | 3.83E-02 | IFNA4 |
| Liver Adhesion | adhesion of hepatic stellate cells | 3.83E-02 | ADAM12 |
| Liver Proliferation | proliferation of hepatic progenitor cells | 3.83E-02 | INHBA |
| Cardiac Damage | reperfusion injury of myocardium | 3.83E-02 | SERPING1 |
| Liver Fibrosis | severe hepatic fibrosis | 3.83E-02 | COL3A1 |
| Cardiac Arrythmia | variation QT syndrome | 3.83E-02 | NHLH1 |
| Liver Necrosis/Cell Death | necrosis of liver | 3.95E-02 | DUSP4,FKBP1A,GADD45B,INHBA,MAP3K8,MYCN,SAA1,SELP,SLC25A5,SLC26A1 |
| Kidney Failure | end stage renal disease | 3.95E-02 | BUB1B,DCN,FKBP1A,MYO1E,PDE5A,VDR |
| Liver Steatosis | nonalcoholic fatty liver disease | 4.46E-02 | ACACA,CPT1A,IL18 |
| Congenital Heart Anomaly | situs inversus totalis | 5.14E-02 | CITED2,LEFTY1 |
| Renal Damage | damage of kidney | 5.33E-02 | BMP7,CYP2C8,IL18,LEPR,RGMB,SELP |
| Liver Damage | injury of liver | 5.44E-02 | AMY2B,COL3A1,FKBP1A,IL18,INHBA,NT5E,SAA1,SERPING1 |
| Renal Atrophy | atrophy of renal tubule | 5.64E-02 | BUB1B,DCN |
| Congenital Heart Anomaly | transposition of great vessels | 5.64E-02 | DVL3,LEFTY1 |
| Cardiac Hypertrophy | hypertrophy of heart septum | 5.69E-02 | GATA4 |
| Renal Inflammation, Renal Nephritis | idiopathic membranous nephropathy | 5.69E-02 | FKBP1A |
| Liver Cholestasis | obstructive jaundice | 5.69E-02 | IL18 |
| Renal Atrophy | atrophy of kidney | 5.92E-02 | BUB1B,DCN,VDR |
| Glomerular Injury, Renal Damage | injury of renal glomerulus | 6.15E-02 | CYP2C8,IL18 |
| Glomerular Injury, Renal Fibrosis | fibrosis of kidney | 6.21E-02 | BMP7,BUB1B,DCN,MYO1E |
| Glomerular Injury, Kidney Failure, Renal Fibrosis | interstitial fibrosis of kidney | 7.23E-02 | BUB1B,DCN,MYO1E |
| Liver Cholestasis | lipopolysaccharide hepatocellular cholestasis | 7.52E-02 | CD68 |
| Congenital Heart Anomaly | mesocardia | 7.52E-02 | CITED2 |
| Cardiac Hypertrophy | hypertrophy of cardiac muscle | 7.90E-02 | GATA4,GATA6,IL18,INHBA,PDE5A,TERT |
| Cardiac Arrythmia | atrial fibrillation | 8.14E-02 | AMY2B,CHI3L1,COL3A1,FKBP1B,TUBD1 |
| Renal Hypoplasia | hypoplasia of kidney | 8.29E-02 | BMP7,DLG1,PBX1 |
| Cardiac Dilation | dilation of heart | 8.36E-02 | GATA4,KLK3 |
| Renal Damage, Renal Tubule Injury | proximal tubular toxicity | 8.54E-02 | COL3A1,HSP90AA1,JUNB,PRKCZ |
| Renal Damage | injury of kidney | 8.83E-02 | BMP7,CYP2C8,IL18,RGMB |
| Cardiac Hypertrophy | hypertrophy of cardiomyocytes | 9.30E-02 | GATA4,GATA6,IL18,INHBA,TERT |
| Glutathione Depletion In Liver, Liver Fibrosis | generation of reactive oxygen species in liver | 9.31E-02 | LEPR |
| Kidney Failure | failure of kidney | 1.02E-01 | AMY2B,BUB1B,DCN,FKBP1A,IL18,MYO1E,PDE5A,VDR |
| Liver Inflammation/Hepatitis | alcoholic hepatitis | 1.08E-01 | COL3A1,PDE5A |
| Congenital Heart Anomaly | bicuspid aortic valve | 1.11E-01 | PBX1 |
| Liver Cholestasis | obstructive cholestasis | 1.11E-01 | CD68 |
| Renal Transformation | transformation of kidney cells | 1.11E-01 | TERT |
| Renal Inflammation, Renal Nephritis | tubular nephritis | 1.11E-01 | DCN |
| Cardiac Arrythmia | ventricular extrasystole | 1.11E-01 | NHLH1 |
| Increased Levels of Red Blood Cells | increased quantity of red blood cells | 1.19E-01 | GATA4,GATA6,INHBA,PBX1 |
| Liver Necrosis/Cell Death | apoptosis of liver cell lines | 1.20E-01 | DUSP4,GADD45B |
| Liver Damage, Liver Inflammation/Hepatitis | chronic active hepatitis | 1.28E-01 | COL3A1 |
| Renal Inflammation, Renal Nephritis | Nephritis | 1.31E-01 | DCN,FKBP1A,GADD45B,GADD45G,SELP,SLC26A1,TP63,VDR |
| Cardiac Congestive Cardiac Failure, Heart Failure | congestive heart failure | 1.46E-01 | FKBP1A,MYCN,PDE5A,ZFPM2 |
| Kidney Failure | ischemic acute renal failure | 1.61E-01 | IL18 |
| Cardiac Proliferation | proliferation of cardiomyocytes | 1.72E-01 | GATA4,GATA6,MYCN |
| Cardiac Damage | injury of heart | 1.74E-01 | INHBA,SERPING1 |
| Cardiac Dysfunction | dysfunction of heart | 1.82E-01 | DUSP4,GATA4,NT5E |
| Nephrosis | nephrosis | 1.82E-01 | FKBP1A,PDE5A,SYNPO |
| Glomerular Injury, Renal Fibrosis | fibrosis of renal glomerulus | 1.93E-01 | BMP7 |
| Liver Hypoplasia | hypoplasia of liver | 2.02E-01 | MYCN,PBX1 |
| Liver Inflammation/Hepatitis | experimental hepatitis | 2.09E-01 | IL18 |
| Liver Necrosis/Cell Death | cell death of hepatocytes | 2.16E-01 | GADD45B,INHBA,MYCN,SAA1,SLC25A5 |
| Renal Damage, Renal Tubule Injury | damage of renal tubule | 2.16E-01 | LEPR,RGMB |
| Renal Inflammation, Renal Nephritis | lupus nephritis | 2.31E-01 | FKBP1A,SELP |
| Cardiac Arteriopathy | coronary artery disease | 2.37E-01 | AMY2B,FKBP1A,KALRN,MCF2L,PDE5A,SELP,TUBD1,VDR |
| Liver Fibrosis | fibrosis of liver | 2.40E-01 | BMP7,COL3A1,NT5E,VDR |
| Liver Necrosis/Cell Death | cell death of liver cells | 2.44E-01 | DUSP4,GADD45B,INHBA,MYCN,SAA1,SLC25A5 |
| Renal Hydronephrosis | hydronephrosis | 2.53E-01 | DCN,DLG1,TNFRSF11B |
| Nephrosis | minimal change nephrotic syndrome | 2.54E-01 | FKBP1A |
| Cardiac Hypoplasia | hypoplasia of heart ventricle | 2.66E-01 | MYCN,ZFPM2 |
| Liver Inflammation/Hepatitis | viral hepatitis | 2.74E-01 | AMY2B,COL3A1,FKBP1A |
| Liver Damage, Liver Inflammation/Hepatitis | chronic hepatitis | 2.84E-01 | AMY2B,COL3A1,FKBP1A |
| Liver Necrosis/Cell Death | apoptosis of hepatocytes | 2.95E-01 | GADD45B,INHBA,MYCN,SAA1 |
| Renal Inflammation, Renal Nephritis | glomerulonephritis | 2.96E-01 | FKBP1A,GADD45B,GADD45G,SELP,VDR |
| Cardiac Inflammation | pericarditis | 2.97E-01 | TUBD1 |
| Renal Necrosis/Cell Death | necrosis of renal tubule | 3.02E-01 | DCN,IL18 |
| Cardiac Necrosis/Cell Death | apoptosis of cardiomyocytes | 3.03E-01 | GATA4,INHBA,LEPR,PPP1R10,TERT |
| Renal Inflammation, Renal Nephritis | autoimmune glomerulonephritis | 3.10E-01 | FKBP1A,VDR |
| Liver Inflammation/Hepatitis | acute hepatitis | 3.10E-01 | COL3A1 |
| Renal Necrosis/Cell Death | apoptosis of renal tubular epithelial cells | 3.10E-01 | DCN |
| Cardiac Fibrosis | fibrosis of myocardium | 3.10E-01 | VDR |
| Increased Levels of ALT | increased localization of ALT | 3.24E-01 | SAA1 |
| Cardiac Inflammation | carditis | 3.45E-01 | IFNA4,TUBD1 |
| Cardiac Arrythmia | arrhythmia of heart ventricle | 3.52E-01 | FKBP1B,NHLH1 |
| Liver Inflammation/Hepatitis, Liver Steatosis | nonalcoholic steatohepatitis | 3.74E-01 | PDE5A |
| Liver Proliferation | proliferation of liver cells | 3.76E-01 | BMP7,CITED2,IL18,INHBA,LEPR |
| Liver Damage, Liver Inflammation/Hepatitis | hepatitis C | 3.80E-01 | AMY2B,FKBP1A |
| Increased Levels of Alkaline Phosphatase | increased activation of alkaline phosphatase | 3.80E-01 | BMP7,TNFRSF11B |
| Cardiac Hypertrophy | hypertrophy of right ventricle | 3.87E-01 | PBX1 |
| Cardiac Fibrosis | interstitial fibrosis of heart | 3.87E-01 | BUB1B |
| Liver Proliferation | proliferation of hepatocytes | 3.88E-01 | BMP7,CITED2,IL18,INHBA |
| Hepatocellular Carcinoma, Liver Hyperplasia/Hyperproliferation | tumorigenesis of hepatocellular carcinoma | 3.98E-01 | BUB1B |
| Cardiac Arrythmia | ventricular fibrillation | 4.10E-01 | NHLH1 |
| Renal Necrosis/Cell Death | apoptosis of kidney cell lines | 4.13E-01 | CDK1,DUSP4,MAP3K8,NLRC4,PAWR,TERT |
| Liver Damage | hepatotoxicity | 4.22E-01 | IL18 |
| Cardiac Infarction | myocardial infarction | 4.22E-01 | COL3A1,CYP2C8,NT5E,SELP,TUBD1 |
| Heart Failure | failure of heart | 4.31E-01 | FKBP1A,GATA4,MYCN,PDE5A,VDR,ZFPM2 |
| Pulmonary Hypertension | Pulmonary Hypertension | 4.41E-01 | IL18,PDE5A |
| Renal Inflammation, Renal Nephritis | IgA nephropathy | 4.55E-01 | VDR |
| Liver Hemorrhaging | bleeding of liver | 4.55E-01 | FKBP1A |
| Heart Failure | chronic heart failure | 4.75E-01 | GATA4 |
| Cardiac Infarction | acute myocardial infarction | 5.04E-01 | SELP,TUBD1 |
| Liver Inflammation/Hepatitis | inflammation of liver | 5.12E-01 | AMY2B,COL3A1,FKBP1A,IL18,PDE5A,PTPRJ |
| Renal Necrosis/Cell Death | cell death of kidney cell lines | 5.15E-01 | CDK1,DUSP4,MAP3K8,NLRC4,PAWR,RGMB,TERT |
| Glomerular Injury, Renal Hypertrophy | hypertrophy of renal glomerulus | 5.15E-01 | LEPR |
| Cardiac Hypoplasia | hypoplasia of trabeculae carne | 5.15E-01 | MYCN |
| Liver Cirrhosis | Cirrhosis | 5.15E-01 | COL3A1,DLG1,NT5E,PDE5A |
| Liver Cirrhosis | primary biliary cirrhosis | 5.22E-01 | COL3A1,DLG1 |
| Cardiac Output | cardiac output | 5.24E-01 | KLK3 |
| Increased Levels of Creatinine | increased quantity of creatinine | 5.34E-01 | IL18 |
| Increased Levels of Hematocrit | increased hematocrit of organism | 5.34E-01 | PBX1,SLC4A4 |
| Renal Necrosis/Cell Death | cell death of kidney cells | 5.38E-01 | CDK1,DCN,DUSP4,MAP3K8,NLRC4,PAWR,RGMB,TERT |
| Pulmonary Hypertension | pulmonary hypertensive arterial disease | 5.52E-01 | PDE5A |
| Liver Hyperplasia/Hyperproliferation | metastasis of liver | 5.60E-01 | MST1R |
| Liver Cirrhosis | cirrhosis of liver | 5.62E-01 | COL3A1,DLG1,NT5E |
| Cardiac Dilation | dilation of left ventricle | 5.69E-01 | MARCKSL1 |
| Renal Proliferation | proliferation of mesangial cells | 5.69E-01 | DCN |
| Liver Fibrosis | activation of hepatic stellate cells | 5.77E-01 | LEPR |
| Cardiac Stenosis | stenosis of aortic valve | 5.93E-01 | BGLAP |
| Liver Fibrosis, Liver Proliferation | proliferation of hepatic stellate cells | 6.17E-01 | LEPR |
| Renal Proliferation | cell proliferation of kidney cell lines | 1.00E+00 | HSP90AA1,NANOG,PFKFB3 |
| Liver Damage, Liver Inflammation/Hepatitis | chronic hepatitis C | 1.00E+00 | AMY2B |
| Liver Steatosis | hepatic steatosis | 1.00E+00 | ACACA,CPT1A,IL18,PDE5A,PRKCDBP |
| Hepatocellular Carcinoma, Liver Hyperplasia/Hyperproliferation | hepatocellular carcinoma | 1.00E+00 | AATK,ACOX3,ACYP2,ADAM12,ALOX12B,ARHGEF4,B4GALT6,BCL11A,BMP7,BRWD3,BUB1B,CADM2,CDKL5,CHD6,CHRNA9,CYP2C8,DCAF12L2,DENND5B,DLEC1,DLG1,DNAJB5,DUSP4,EDIL3,EMILIN3,ESPN,ESRRB,ETV1,FAM46C,FILIP1,FKBP1A,FKBP1B,FOXQ1,GALNT7,GPSM2,GRAMD1B,GTF3C1,HERC1,HSP90AA1,HYDIN,IL18,INHBA,IQSEC3,JMJD1C,KALRN,KCNAB1,KIDINS220,KIF3C,LEFTY1,LEPR,MAPKAP1,MDFIC,MEX3B,MST1R,MYO18B,MYO1E,NDUFV2,NFX1,NLRC4,NOTUM,NR1D1,NT5E,PLXNB1,PRIM2,PTPN13,PTPRE,RAPSN,RBM47,RGMB,RGS12,RNF130,RNF39,RREB1,SCAF8,SELP,SERPINA10,SERPING1,SH3PXD2A,SKIL,SLC1A4,SLC26A1,SLC45A3,SNRK,SNTG2,SPON1,SPRY4,SYNPO,TM6SF2,TUBD1,UNCX,USP15,USP19,VDR,WBSCR22,WWP1,ZFPM2,ZNF175,ZNF282 |
| Liver Hyperplasia/Hyperproliferation | liver cancer | 1.00E+00 | AATK,ACOX3,ACYP2,ADAM12,ALOX12B,ARHGEF4,B4GALT6,BCL11A,BMP7,BRWD3,BUB1B,CADM2,CDKL5,CHD6,CHRNA9,CYP2C8,DCAF12L2,DENND5B,DLEC1,DLG1,DNAJB5,DUSP4,EDIL3,EMILIN3,ESPN,ESRRB,ETV1,FAM46C,FILIP1,FKBP1A,FKBP1B,FOXQ1,GALNT7,GPSM2,GRAMD1B,GTF3C1,HERC1,HSP90AA1,HYDIN,IL18,INHBA,IQSEC3,JMJD1C,KALRN,KCNAB1,KIDINS220,KIF3C,LEFTY1,LEPR,MAPKAP1,MDFIC,MEX3B,MST1R,MYO18B,MYO1E,NDUFV2,NFX1,NLRC4,NOTUM,NR1D1,NT5E,PDE5A,PHGDH,PLXNB1,PRIM2,PTPN13,PTPRE,RAPSN,RBM47,RGMB,RGS12,RNF130,RNF39,RREB1,SCAF8,SELP,SERPINA10,SERPING1,SH3PXD2A,SKIL,SLC1A4,SLC26A1,SLC45A3,SNRK,SNTG2,SPON1,SPRY4,SYNPO,TM6SF2,TUBD1,UNCX,USP15,USP19,VDR,WBSCR22,WWP1,ZFPM2,ZNF175,ZNF282 |
| Liver Hyperplasia/Hyperproliferation | liver tumor | 1.00E+00 | AATK,ACOX3,ACYP2,ADAM12,ALOX12B,ARHGEF4,B4GALT6,BCL11A,BMP7,BRWD3,BUB1B,CADM2,CDKL5,CHD6,CHRNA9,CYP2C8,DCAF12L2,DENND5B,DLEC1,DLG1,DNAJB5,DUSP4,EDIL3,EMILIN3,ESPN,ESRRB,ETV1,FAM46C,FILIP1,FKBP1A,FKBP1B,FOXQ1,GALNT7,GPSM2,GRAMD1B,GTF3C1,HERC1,HSP90AA1,HYDIN,IL18,INHBA,IQSEC3,JMJD1C,KALRN,KCNAB1,KIDINS220,KIF3C,LEFTY1,LEPR,MAPKAP1,MDFIC,MEX3B,MST1R,MYO18B,MYO1E,NDUFV2,NFX1,NLRC4,NOTUM,NR1D1,NT5E,PAWR,PDE5A,PHGDH,PLXNB1,PRIM2,PTPN13,PTPRE,RAPSN,RBM47,RGMB,RGS12,RNF130,RNF39,RREB1,SCAF8,SELP,SERPINA10,SERPING1,SH3PXD2A,SKIL,SLC1A4,SLC26A1,SLC45A3,SNRK,SNTG2,SPON1,SPRY4,SYNPO,TERT,TM6SF2,TUBD1,UNCX,USP15,USP19,VDR,WBSCR22,WWP1,ZFPM2,ZNF175,ZNF282 |

**Table S6 Disease and function of SMI VS eNOS KO.**

| **Categories** | **Diseases or Functions Annotation** | **p-Value** | Molecules |
| --- | --- | --- | --- |
| Cardiac Dysfunction | systolic dysfunction | 9.59E-05 | ACE,CACNA1F,CACNB4,FKBP1A,MARCKSL1,PDE5A |
| Congenital Heart Anomaly | heart septal defect | 1.92E-04 | DVL3,EDN1,FKBP1A,LEFTY1,LIN28A,MYH6,PBX1,RGS19,TBX20,VEGFA,ZFPM2 |
| Congenital Heart Anomaly | familial congenital heart disease | 2.63E-04 | DVL3,EDN1,GATA5,LEFTY1,MYH6,PATZ1,PBX1,TBX20,VEGFA,ZFPM2 |
| Congenital Heart Anomaly | familial cardiac septal defect | 4.31E-04 | DVL3,EDN1,LEFTY1,MYH6,PBX1,TBX20,ZFPM2 |
| Congenital Heart Anomaly | ventricular septal defect | 4.52E-04 | DVL3,EDN1,FKBP1A,LEFTY1,LIN28A,PBX1,RGS19,TBX20,VEGFA,ZFPM2 |
| Congenital Heart Anomaly | congenital heart disease | 1.27E-03 | DVL3,EDN1,FKBP1A,GATA5,LEFTY1,LIN28A,MYH6,NOTCH1,PATZ1,PBX1,RGS19,TBX20,VEGFA,ZFPM2 |
| Cardiac Dysfunction, Heart Failure | systolic heart failure | 1.75E-03 | ACE,CACNA1F,CACNB4 |
| Renal Inflammation, Renal Nephritis | idiopathic membranous nephropathy | 1.93E-03 | FKBP1A,NR3C1 |
| Congenital Heart Anomaly | familial ventricular septal defect | 2.05E-03 | DVL3,EDN1,LEFTY1,PBX1,TBX20,ZFPM2 |
| Renal Hydronephrosis | hydronephrosis | 2.15E-03 | ACE,DACT1,DCN,DLG1,FGF13,FSTL1,HIVEP3,TNFRSF11B |
| Cardiac Arrythmia | supraventricular arrhythmia | 2.78E-03 | ACE,CACNA1F,CACNB4,CHRM2,COL3A1,KCNA7,KCNJ12,MYH6,NR3C1,VEGFA |
| Congenital Heart Anomaly | conotruncal heart malformations | 3.54E-03 | DVL3,EDN1,LEFTY1,PATZ1,PBX1,VEGFA,ZFPM2 |
| Cardiac Arrythmia | arrhythmia | 3.57E-03 | ACE,CACNA1F,CACNB4,CHRM2,COL3A1,KCNA7,KCNJ12,MMP2,MYH6,NR3C1,Pde4d,SLC2A5,VEGFA |
| Congenital Heart Anomaly | atresia of tricuspid valve | 3.79E-03 | LEFTY1,ZFPM2 |
| Liver Cholestasis | lipopolysaccharide hepatocellular cholestasis | 3.79E-03 | CD68,GJB2 |
| Cardiac Transformation | transformation of endocardial cells | 3.79E-03 | MMP2,VEGFA |
| Nephrosis | minimal change nephrotic syndrome | 6.03E-03 | CDKN1A,FKBP1A,NR3C1 |
| Pulmonary Hypertension | primary pulmonary hypertension | 7.28E-03 | CAV1,EDN1,KDR |
| Liver Proliferation | proliferation of liver cells | 7.48E-03 | BMP7,C3AR1,CAV1,CDKN1A,COL1A1,EDN1,FGF7,IL18,LEPR,S1PR2,SKP2,VEGFA |
| Congenital Heart Anomaly | double outlet right ventricle | 8.13E-03 | DVL3,EDN1,LEFTY1,PBX1,ZFPM2 |
| Liver Damage | injury of liver | 8.64E-03 | CD40,CDKN1A,COL3A1,DDR2,DDX5,EBI3,EDN1,FKBP1A,IL18,NR3C1,NT5E,SERPING1 |
| Congenital Heart Anomaly | bicuspid aortic valve | 9.16E-03 | GATA5,PBX1 |
| Liver Cholestasis | obstructive cholestasis | 9.16E-03 | CD68,GJB2 |
| Renal Hypoplasia | hypoplasia of kidney | 1.13E-02 | ACE,BMP7,DLG1,FGF7,PBX1 |
| Renal Inflammation, Renal Nephritis | membranous glomerulonephritis | 1.19E-02 | CDKN1A,FKBP1A,NR3C1 |
| Liver Damage, Liver Inflammation/Hepatitis | chronic active hepatitis | 1.26E-02 | COL3A1,NR3C1 |
| Cardiac Inflammation | myocarditis | 1.31E-02 | BCL6,CCL3L3,IFNA4,MYH6 |
| Renal Atrophy | atrophy of renal tubule | 1.37E-02 | ACE,DCN,MMP2 |
| Congenital Heart Anomaly | transposition of great vessels | 1.37E-02 | DVL3,LEFTY1,PATZ1 |
| Congenital Heart Anomaly | familial atrial septal defect | 1.65E-02 | MYH6,TBX20 |
| Cardiac Congestive Cardiac Failure, Heart Failure | congestive heart failure | 1.83E-02 | ACE,CACNA1F,CAV1,FKBP1A,NR3C1,PDE5A,ZFPM2 |
| Liver Damage | damage of liver | 1.99E-02 | C3AR1,CD40,CDKN1A,COL3A1,DDR2,DDX5,EBI3,EDN1,FKBP1A,IL18,NR3C1,NT5E,SERPING1 |
| Cardiac Hypertrophy | ventricular hypertrophy | 2.03E-02 | ACE,CAV1,EDN1,FKBP1A,IL18,NT5E,PBX1,PDE5A |
| Pulmonary Hypertension | pulmonary hypertensive arterial disease | 2.03E-02 | CAV1,EDN1,KDR,PDE5A |
| Cardiac Inflammation | carditis | 2.24E-02 | BCL6,CCL3L3,IFNA4,MYH6,NR3C1 |
| Cardiac Dilation | dilation of left ventricle | 2.38E-02 | ADCY5,CAV1,MARCKSL1,MYH6 |
| Cardiac Arrythmia | arrhythmia of heart ventricle | 2.38E-02 | CACNA1F,CACNB4,CHRM2,KCNA7,KCNJ12 |
| Cardiac Inflammation | acute rheumatic carditis | 2.56E-02 | NR3C1 |
| Congenital Heart Anomaly | atrial septal defect 4 | 2.56E-02 | TBX20 |
| Congenital Heart Anomaly | atrial septal defect type 3 | 2.56E-02 | MYH6 |
| Cardiac Inflammation | chronic inflammation of heart | 2.56E-02 | IFNA4 |
| Glomerular Injury | collapsing glomerulopathy | 2.56E-02 | VEGFA |
| Decreased Levels of Albumin | decreased accumulation of albumin | 2.56E-02 | CAV1 |
| Glomerular Injury | delayed hypersensitive reaction of renal glomerulus | 2.56E-02 | IL18 |
| Glomerular Injury, Renal Hyperplasia/Hyperproliferation | hyperplasia of parietal epithelial cells | 2.56E-02 | CDKN1A |
| Pulmonary Hypertension | hypertension of pulmonary artery | 2.56E-02 | CAV1 |
| Cardiac Hypertrophy | hypertrophy of cardiac anterior wall | 2.56E-02 | MYH6 |
| Cardiac Hypoplasia | hypoplasia of ventricular compact zone | 2.56E-02 | ZFPM2 |
| Increased Levels of Albumin | increased permeation of albumin | 2.56E-02 | VEGFA |
| Increased Levels of Alkaline Phosphatase | increased production of alkaline phosphatase | 2.56E-02 | VEGFA |
| Renal Inflammation, Renal Nephritis | necrotizing glomerulonephritis | 2.56E-02 | VEGFA |
| Pulmonary Hypertension | primary pulmonary hypertension 3 | 2.56E-02 | CAV1 |
| Cardiac Necrosis/Cell Death | quantity of apoptotic ventricular myocytes | 2.56E-02 | MMP2 |
| Cardiac Stenosis | retinal arterial macroaneurysm with supravalvular pulmonic stenosis | 2.56E-02 | IGFBP7 |
| Cardiac Arrythmia | sick sinus syndrome type 3 | 2.56E-02 | MYH6 |
| Liver Cirrhosis | stage 3 primary biliary cirrhosis | 2.56E-02 | COL3A1 |
| Liver Cirrhosis | stage 4 primary biliary cirrhosis | 2.56E-02 | COL3A1 |
| Cardiac Infarction | myocardial infarction | 2.66E-02 | ACE,C6,CCL3L3,COL3A1,DUSP6,EDN1,FSTL1,KCNA7,KCNJ12,NT5E,VEGFA |
| Liver Cirrhosis | Cirrhosis | 2.72E-02 | CHRM2,COL1A1,COL3A1,DLG1,IL7R,MMP2,NOTCH1,NR3C1,NT5E,PDE5A |
| Congenital Heart Anomaly | atrial septal defect | 2.76E-02 | LEFTY1,MYH6,TBX20,ZFPM2 |
| Cardiac Arrythmia | atrial fibrillation | 3.34E-02 | ACE,CHRM2,COL3A1,KCNA7,KCNJ12,NR3C1,VEGFA |
| Renal Inflammation, Renal Nephritis | Nephritis | 3.52E-02 | ACE,CCL3L3,CDKN1A,DCN,FKBP1A,GADD45G,KDR,NR3C1,SLC26A1,TNFSF13B,TP63,VEGFA |
| Cardiac Necrosis/Cell Death | cell death of ventricular myocytes | 3.72E-02 | CDKN1A,FSTL1,MMP2 |
| Liver Cirrhosis | cirrhosis of liver | 4.07E-02 | CHRM2,COL3A1,DLG1,IL7R,MMP2,NOTCH1,NR3C1,NT5E |
| Cardiac Arteriopathy | variant angina | 4.23E-02 | CACNA1F,CACNB4 |
| Nephrosis | nephrosis | 4.52E-02 | CA7,CDKN1A,FKBP1A,NR3C1,PDE5A |
| Pulmonary Hypertension | Pulmonary Hypertension | 4.74E-02 | CAV1,EDN1,IL18,KDR,PDE5A |
| Heart Failure | failure of heart | 4.80E-02 | ACE,CA7,CACNA1F,CACNB4,CAV1,DUSP6,FKBP1A,NR3C1,Pde4d,PDE5A,VEGFA,ZFPM2 |
| Renal Damage, Renal Tubule Injury | damage of tubulointerstitium | 4.86E-02 | ACE,EDN1 |
| Cardiac Arteriopathy | coronary artery disease | 5.00E-02 | ACE,CACNA1F,CACNB4,DOCK4,EDN1,FKBP1A,KALRN,KDR,LRRC17,NR3C1,PDE5A,RBFOX1,VEGFA |
| Kidney Failure | failure of kidney | 5.01E-02 | ACE,CDKN1A,DCN,EDN1,FKBP1A,IL18,MMP2,NR3C1,PDE5A,SKP2,VEGFA |
| Cardiac Inflammation | acute myocarditis | 5.05E-02 | IFNA4 |
| Renal Inflammation, Renal Nephritis | acute pyelonephritis | 5.05E-02 | KDR |
| Liver Adhesion | adhesion of hepatic stellate cells | 5.05E-02 | ADAM12 |
| Liver Edema | edema of liver | 5.05E-02 | VEGFA |
| Liver Cholestasis | ethynylestradiol hepatocellular cholestasis | 5.05E-02 | GJB2 |
| Glomerular Injury | glomerular endotheliosis | 5.05E-02 | VEGFA |
| Increased Levels of Albumin | increased flux of albumin | 5.05E-02 | KDR |
| Renal Regeneration | regeneration of glomerular capillary | 5.05E-02 | VEGFA |
| Cardiac Damage | reperfusion injury of myocardium | 5.05E-02 | SERPING1 |
| Liver Fibrosis | severe hepatic fibrosis | 5.05E-02 | COL3A1 |
| Cardiac Arteriopathy | stable coronary artery disease | 5.05E-02 | ACE |
| Kidney Failure | chronic renal failure | 5.07E-02 | ACE,CDKN1A,DCN,FKBP1A,MMP2,PDE5A,SKP2,VEGFA |
| Renal Inflammation, Renal Nephritis | crescentic glomerulonephritis | 5.51E-02 | CCL3L3,VEGFA |
| Cardiac Arrythmia, Tachycardia | hemodynamically unstable ventricular tachycardia | 5.51E-02 | KCNA7,KCNJ12 |
| Liver Hyperplasia/Hyperproliferation | hyperplasia of liver | 5.51E-02 | SKP2,SPI1 |
| Cardiac Arrythmia | recurrent ventricular fibrillation | 5.51E-02 | KCNA7,KCNJ12 |
| Cardiac Arrythmia | atrial flutter | 5.56E-02 | ACE,CHRM2,KCNJ12 |
| Congenital Heart Anomaly | persistent truncus arteriosus | 5.56E-02 | DVL3,PBX1,VEGFA |
| Cardiac Arrythmia, Tachycardia | tachycardia | 5.67E-02 | CACNA1F,CACNB4,CHRM2,KCNA7,KCNJ12 |
| Liver Proliferation | proliferation of hepatocytes | 5.87E-02 | BMP7,CAV1,CDKN1A,FGF7,IL18,S1PR2,SKP2,VEGFA |
| Renal Inflammation, Renal Nephritis | autoimmune glomerulonephritis | 6.10E-02 | ACE,CDKN1A,FKBP1A,NR3C1 |
| Cardiac Hypertrophy | hypertrophy of left ventricle | 6.17E-02 | ACE,CAV1,FKBP1A,NT5E,PBX1 |
| Cardiac Inflammation | experimental autoimmune myocarditis | 6.19E-02 | CCL3L3,MYH6 |
| Glomerular Injury, Renal Hypertrophy | hypertrophy of renal glomerulus | 6.83E-02 | EDN1,LEPR,VEGFA |
| Renal Damage, Renal Tubule Injury | proximal tubular toxicity | 6.97E-02 | CCL3L3,COL3A1,COL5A1,GJB2,HSP90AA1 |
| Cardiac Hypertrophy | hypertrophy of heart | 7.13E-02 | ACE,CAV1,CDKN1A,CNOT3,DACT1,EDN1,FKBP1A,IL18,LEPR,MYH6,NT5E,PBX1,PDE5A,TCF15,TRPC1 |
| Liver Proliferation | arrest in growth of hepatocytes | 7.49E-02 | SKP2 |
| Cardiac Inflammation | inflammatory cardiomyopathy | 7.49E-02 | NR3C1 |
| Cardiac Infarction | nonfatal myocardial infarction | 7.49E-02 | ACE |
| Liver Cholestasis | obstruction of bile duct | 7.49E-02 | GJB2 |
| Liver Cholestasis | obstructive jaundice | 7.49E-02 | IL18 |
| Liver Cirrhosis | primary biliary cirrhosis | 8.11E-02 | COL3A1,DLG1,IL7R,NOTCH1,NR3C1 |
| Liver Inflammation/Hepatitis | acute hepatitis | 8.40E-02 | COL3A1,MMP2 |
| Cardiac Inflammation | inflammation of heart | 8.70E-02 | BCL6,CAV1,IFNA4 |
| Renal Hypoplasia | hypoplasia of renal cortex | 9.85E-02 | FGF7 |
| Renal Hypoplasia | hypoplasia of renal medulla | 9.85E-02 | ACE |
| Cardiac Necrosis/Cell Death | necrosis of myocardium | 9.85E-02 | MYH6 |
| Renal Dysplasia | renal tubular dysgenesis | 9.85E-02 | ACE |
| Renal Damage, Renal Tubule Injury | damage of renal tubule | 1.08E-01 | ACE,EDN1,LEPR |
| Congenital Heart Anomaly | atrioventricular septal defect and common atrioventricular junction | 1.08E-01 | LEFTY1,ZFPM2 |
| Renal Inflammation, Renal Nephritis | glomerulonephritis | 1.08E-01 | ACE,CCL3L3,CDKN1A,FKBP1A,GADD45G,NR3C1,TNFSF13B,VEGFA |
| Cardiac Necrosis/Cell Death | apoptosis of cardiomyocytes | 1.13E-01 | ADCY5,CDKN1A,EDN1,FSTL1,LEPR,MMP2,TBX20,VEGFA |
| Kidney Failure | end stage renal disease | 1.14E-01 | ACE,DCN,FKBP1A,MMP2,PDE5A,SKP2 |
| Liver Hyperplasia/Hyperproliferation | liver cancer | 1.18E-01 | AATK,ABCA5,ABCG4,ACE,ACOX3,ADAM12,ADCY5,ALOX12B,ANGPTL1,APC2,ARHGAP17,BMP7,BRAT1,BRWD3,C3AR1,CACNB4,CADM2,CBX7,CCDC80,CCNL1,CD22,CDC25B,CDCA3,CDH22,CDKN1A,CDKN2B,CHD6,CHRM2,CKS2,CNOT3,COL1A1,COL5A1,CPEB4,CPLX3,DACT1,DCLK1,DDR2,DENND5B,DLEC1,DLG1,DOCK4,DUSP4,EFHC1,EMILIN3,ESPN,EVI2A,FCRL5,FGF12,FGF13,FHOD3,FKBP1A,FOXQ1,FRK,GALNT7,GATA5,GLTP,GPSM2,GRAMD1B,GTF3C1,HERC1,HGFAC,HIVEP3,HLX,HSP90AA1,HYDIN,IGSF10,IL18,IL7R,IQGAP2,IRX1,IRX2,JMJD1C,KALRN,KCNJ12,KDR,KHSRP,KIDINS220,KIF20B,KIF3C,KLHL3,KRTAP19-3,LEFTY1,LEPR,LIN28A,MAPKAP1,MBNL3,MDFIC,MEX3B,MGEA5,MMP2,MMP27,MPP2,MYH6,MYL4,MYO18B,MYO1F,NDUFV2,NEK6,NFKBIE,NFX1,NKD2,NLRC4,NOTCH1,NOTUM,NR1D1,NR3C1,NT5E,PAMR1,PCLO,PDE5A,PHGDH,PIF1,POGK,PRDM5,PRIM2,PTPN5,RAPSN,RASAL2,RBM47,RGS9BP,RHOBTB1,RNF130,RNF39,SCAF8,SERPING1,SERTAD4,SGK2,SH3PXD2A,SHC4,SIK2,SKIL,SKP2,SLC26A1,SLC2A5,SLC45A3,SNRK,SNTG2,SPON1,STX11,SYT15,TBX20,TM4SF5,TM6SF2,TTC17,TTC25,UNCX,USP15,VCAN,VEGFA,WBSCR22,WDFY3,WWP1,ZDHHC23,ZFPM2,ZNF282,ZNF471,ZNF521 |
| Liver Hyperplasia/Hyperproliferation | liver tumor | 1.20E-01 | AATK,ABCA5,ABCG4,ACE,ACOX3,ADAM12,ADCY5,ALOX12B,ANGPTL1,APC2,ARHGAP17,BMP7,BRAT1,BRWD3,C3AR1,CACNB4,CADM2,CBX7,CCDC80,CCNL1,CD22,CDC25B,CDCA3,CDH22,CDKN1A,CDKN2B,CHD6,CHRM2,CKS2,CNOT3,COL1A1,COL5A1,CPEB4,CPLX3,CYP1B1,DACT1,DCLK1,DDR2,DENND5B,DLEC1,DLG1,DOCK4,DUSP4,EFHC1,EMILIN3,ESPN,EVI2A,FCRL5,FGF12,FGF13,FHOD3,FKBP1A,FOXQ1,FRK,GALNT7,GATA5,GLTP,GPSM2,GRAMD1B,GTF3C1,HERC1,HGFAC,HIVEP3,HLX,HSP90AA1,HYDIN,IGSF10,IL18,IL7R,IQGAP2,IRX1,IRX2,JMJD1C,KALRN,KCNJ12,KDR,KHSRP,KIDINS220,KIF20B,KIF3C,KLHL3,KRTAP19-3,LEFTY1,LEPR,LIN28A,MAPKAP1,MBNL3,MDFIC,MEX3B,MGEA5,MMP2,MMP27,MPP2,MYH6,MYL4,MYO18B,MYO1F,NDUFV2,NEK6,NFKBIE,NFX1,NKD2,NLRC4,NOTCH1,NOTUM,NR1D1,NR3C1,NT5E,PAMR1,PCLO,PDE5A,PHGDH,PIF1,POGK,PRDM5,PRIM2,PTPN5,RAPSN,RASAL2,RBM47,RGS9BP,RHOBTB1,RNF130,RNF39,SCAF8,SERPING1,SERTAD4,SGK2,SH3PXD2A,SHC4,SIK2,SKIL,SKP2,SLC26A1,SLC2A5,SLC45A3,SNRK,SNTG2,SPON1,STX11,SYT15,TBX20,TM4SF5,TM6SF2,TTC17,TTC25,UNCX,USP15,VCAN,VEGFA,WBSCR22,WDFY3,WWP1,ZDHHC23,ZFPM2,ZNF282,ZNF471,ZNF521 |
| Hepatocellular Carcinoma, Liver Hyperplasia/Hyperproliferation | hepatocellular carcinoma | 1.20E-01 | AATK,ABCA5,ABCG4,ACE,ACOX3,ADAM12,ADCY5,ALOX12B,ANGPTL1,APC2,ARHGAP17,BMP7,BRAT1,BRWD3,C3AR1,CACNB4,CADM2,CBX7,CCDC80,CCNL1,CD22,CDC25B,CDCA3,CDH22,CDKN1A,CDKN2B,CHD6,CHRM2,CKS2,CNOT3,COL1A1,COL5A1,CPEB4,CPLX3,DACT1,DCLK1,DDR2,DENND5B,DLEC1,DLG1,DOCK4,DUSP4,EFHC1,EMILIN3,ESPN,EVI2A,FCRL5,FGF12,FGF13,FHOD3,FKBP1A,FOXQ1,FRK,GALNT7,GATA5,GLTP,GPSM2,GRAMD1B,GTF3C1,HERC1,HGFAC,HIVEP3,HLX,HSP90AA1,HYDIN,IGSF10,IL18,IL7R,IQGAP2,IRX1,IRX2,JMJD1C,KALRN,KCNJ12,KDR,KHSRP,KIDINS220,KIF20B,KIF3C,KLHL3,KRTAP19-3,LEFTY1,LEPR,LIN28A,MAPKAP1,MBNL3,MDFIC,MEX3B,MGEA5,MMP2,MMP27,MPP2,MYH6,MYL4,MYO18B,MYO1F,NDUFV2,NEK6,NFKBIE,NFX1,NKD2,NLRC4,NOTCH1,NOTUM,NR1D1,NR3C1,NT5E,PAMR1,PCLO,PIF1,POGK,PRDM5,PRIM2,PTPN5,RAPSN,RASAL2,RBM47,RGS9BP,RHOBTB1,RNF130,RNF39,SCAF8,SERPING1,SERTAD4,SGK2,SH3PXD2A,SHC4,SIK2,SKIL,SKP2,SLC26A1,SLC2A5,SLC45A3,SNRK,SNTG2,SPON1,STX11,SYT15,TBX20,TM4SF5,TM6SF2,TTC17,TTC25,UNCX,USP15,VCAN,WBSCR22,WDFY3,WWP1,ZDHHC23,ZFPM2,ZNF282,ZNF471,ZNF521 |
| Renal Proliferation | proliferation of kidney cells | 1.21E-01 | CDKN1A,DCN,EDN1,SKP2 |
| Liver Damage, Liver Inflammation/Hepatitis | chronic autoimmune hepatitis | 1.22E-01 | NR3C1 |
| Renal Inflammation, Renal Nephritis | experimental crescentic glomerulonephritis | 1.22E-01 | VEGFA |
| Glutathione Depletion In Liver, Liver Fibrosis | generation of reactive oxygen species in liver | 1.22E-01 | LEPR |
| Decreased Levels of Albumin | increased uptake of albumin | 1.22E-01 | CAV1 |
| Liver Fibrosis | mitogenesis of hepatic stellate cells | 1.22E-01 | VEGFA |
| Cardiac Hypertrophy | hypertrophy of ventricular myocytes | 1.25E-01 | EDN1,IL18 |
| Liver Fibrosis, Liver Proliferation | proliferation of hepatic stellate cells | 1.30E-01 | COL1A1,EDN1,LEPR |
| Cardiac Necrosis/Cell Death | necrosis of cardiac muscle | 1.32E-01 | ADCY5,CDKN1A,EDN1,FSTL1,LEPR,MMP2,MYH6,TBX20,VEGFA |
| Cardiac Necrosis/Cell Death | apoptosis of ventricular myocytes | 1.34E-01 | FSTL1,MMP2 |
| Cardiac Hypertrophy | hypertrophy of right ventricle | 1.34E-01 | CAV1,PBX1 |
| Glomerular Injury, Renal Fibrosis | fibrosis of kidney | 1.35E-01 | BMP7,DCN,MMP2,SKP2 |
| Glomerular Injury, Kidney Failure, Renal Fibrosis | interstitial fibrosis of kidney | 1.36E-01 | DCN,MMP2,SKP2 |
| Hepatocellular Carcinoma, Liver Hyperplasia/Hyperproliferation | incidence of hepatocellular carcinoma | 1.42E-01 | CBX7,CDKN1A,IQGAP2 |
| Glomerular Injury, Renal Necrosis/Cell Death | apoptosis of podocytes | 1.42E-01 | CDKN1A,EDN1 |
| Cardiac Damage | damage of cardiac muscle | 1.42E-01 | CAV1,SERPING1 |
| Cardiac Arrythmia, Tachycardia | paroxysmal supraventricular tachycardia | 1.42E-01 | CACNA1F,CACNB4 |
| Congenital Heart Anomaly | double aortic arch | 1.44E-01 | VEGFA |
| Renal Transformation | transformation of kidney cells | 1.44E-01 | NOTCH1 |
| Renal Inflammation, Renal Nephritis | tubular nephritis | 1.44E-01 | DCN |
| Renal Damage | damage of kidney | 1.47E-01 | ACE,BMP7,C3AR1,EDN1,IL18,LEPR |
| Renal Proliferation | proliferation of glomerular cells | 1.54E-01 | CDKN1A,DCN,EDN1 |
| Cardiac Enlargement | enlargement of cardiomyocytes | 1.66E-01 | EDN1 |
| Renal Proliferation | proliferation of podocytes | 1.66E-01 | CDKN1A |
| Renal Proliferation | proliferation of renal tubular epithelial cells | 1.66E-01 | SKP2 |
| Hepatocellular Carcinoma, Liver Hyperplasia/Hyperproliferation | quantity of hepatocellular carcinoma | 1.66E-01 | CDKN1A |
| Kidney Failure | acute renal failure | 1.66E-01 | ACE,EDN1,IL18 |
| Cardiac Arrythmia, Tachycardia | ventricular tachycardia | 1.66E-01 | CHRM2,KCNA7,KCNJ12 |
| Liver Inflammation/Hepatitis | alcoholic hepatitis | 1.69E-01 | COL3A1,PDE5A |
| Liver Hyperplasia/Hyperproliferation | liver adenoma | 1.79E-01 | CBX7,CYP1B1 |
| Renal Necrosis/Cell Death | necrosis of renal tubule | 1.79E-01 | CDKN1A,DCN,IL18 |
| Renal Inflammation, Renal Nephritis | acute phase crescentic glomerulonephritis | 1.87E-01 | CCL3L3 |
| Cardiac Proliferation | proliferation of ventricular myocytes | 1.87E-01 | EDN1 |
| Renal Inflammation, Renal Nephritis | IgA nephropathy | 1.88E-01 | ACE,NR3C1 |
| Heart Failure | chronic heart failure | 2.07E-01 | ACE,CA7 |
| Cardiac Infarction | infarction of heart | 2.08E-01 | CAV1 |
| Kidney Failure | ischemic acute renal failure | 2.08E-01 | IL18 |
| Cardiac Stenosis | non-rheumatic aortic stenosis | 2.08E-01 | MMP2 |
| Hepatocellular Carcinoma, Liver Hyperplasia/Hyperproliferation | size of hepatocellular carcinoma | 2.08E-01 | IQGAP2 |
| Congenital Heart Anomaly | tetralogy of Fallot | 2.08E-01 | ZFPM2 |
| Increased Levels of Hematocrit | increased hematocrit of organism | 2.10E-01 | ACE,PBX1,SLC4A4,SPI1 |
| Renal Proliferation | cell proliferation of kidney cell lines | 2.15E-01 | CDKN1A,DUSP6,HSP90AA1,IQGAP2,NANOG,PFKFB3,SKP2 |
| Liver Inflammation/Hepatitis | viral hepatitis | 2.21E-01 | COL3A1,DDX5,FKBP1A,NR3C1 |
| Glomerular Injury | glomerulosclerosis | 2.22E-01 | BMP7,DCN,MMP2,SKP2,VEGFA |
| Cardiac Pulmonary Embolism | acute pulmonary embolism | 2.28E-01 | VEGFA |
| Renal Necrosis/Cell Death | apoptosis of proximal tubule cells | 2.28E-01 | CDKN1A |
| Renal Atrophy | atrophy of renal medulla | 2.28E-01 | ACE |
| Cardiac Hyperplasia/Hyperproliferation | hyperplasia of heart | 2.28E-01 | DUSP6 |
| Hepatocellular Carcinoma, Liver Hyperplasia/Hyperproliferation | inoperable hepatocellular carcinoma | 2.28E-01 | KDR |
| Hepatocellular Carcinoma, Liver Hyperplasia/Hyperproliferation | local hepatocellular carcinoma | 2.28E-01 | KDR |
| Hepatocellular Carcinoma, Liver Hyperplasia/Hyperproliferation | metastatic hepatocellular carcinoma | 2.28E-01 | KDR |
| Liver Inflammation/Hepatitis | inflammation of liver | 2.29E-01 | BCL6,CCL3L3,COL3A1,DDX5,FKBP1A,IL18,MMP2,NR3C1,PDE5A,SPI1 |
| Liver Damage, Liver Inflammation/Hepatitis | chronic hepatitis | 2.32E-01 | COL3A1,DDX5,FKBP1A,NR3C1 |
| Renal Necrosis/Cell Death | apoptosis of tubular cells | 2.35E-01 | CDKN1A,DCN |
| Liver Fibrosis | fibrosis of liver | 2.36E-01 | BMP7,COL1A1,COL3A1,NT5E,S1PR2 |
| Renal Atrophy | atrophy of renal cortex | 2.48E-01 | ACE |
| Glomerular Injury, Renal Fibrosis | fibrosis of renal glomerulus | 2.48E-01 | BMP7 |
| Hepatocellular Carcinoma, Liver Hyperplasia/Hyperproliferation | unresectable hepatocellular carcinoma | 2.48E-01 | KDR |
| Increased Levels of Alkaline Phosphatase | increased activation of alkaline phosphatase | 2.53E-01 | BMP7,NOTCH1,TNFRSF11B |
| Cardiac Output | cardiac output | 2.54E-01 | KLK3,VEGFA |
| Liver Inflammation/Hepatitis | experimental hepatitis | 2.68E-01 | IL18 |
| Cardiac Arrythmia | long-QT syndrome | 2.68E-01 | SLC2A5 |
| Liver Steatosis | nonalcoholic fatty liver disease | 2.83E-01 | ACE,IL18 |
| Renal Dilation | dilation of renal tubule | 2.86E-01 | SKP2 |
| Glomerular Injury, Renal Hypertrophy | hypertrophy of mesangial cells | 2.86E-01 | EDN1 |
| Renal Necrosis/Cell Death | cell viability of kidney cell lines | 2.94E-01 | BCL6,CAV1,MAP3K8 |
| Liver Hypoplasia | hypoplasia of liver | 3.02E-01 | HLX,PBX1 |
| Renal Proliferation | proliferation of mesangial cells | 3.02E-01 | DCN,EDN1 |
| Cardiac Damage, Cardiac Degeneration | degeneration of cardiomyocytes | 3.05E-01 | CAV1 |
| Kidney Failure, Liver Failure | hepatorenal syndrome | 3.05E-01 | EDN1 |
| Liver Hyperplasia/Hyperproliferation | progressive liver metastasis | 3.05E-01 | KDR |
| Liver Hyperplasia/Hyperproliferation | symptomatic stage iodine-refractory liver metastasis | 3.05E-01 | KDR |
| Cardiac Fibrosis | fibrosis of heart | 3.10E-01 | ACE,BCL6,CAV1,CNOT3,MYH6,SNAI1 |
| Liver Fibrosis | activation of hepatic stellate cells | 3.11E-01 | EDN1,LEPR |
| Heart Failure | cardiac decompensation | 3.22E-01 | DUSP6 |
| Glutathione Depletion In Liver | conjugation of glutathione | 3.22E-01 | CYP1B1 |
| Increased Levels of AST | increased localization of AST | 3.22E-01 | MAP3K8 |
| Increased Levels of LDH | increased release of LDH | 3.22E-01 | EDN1 |
| Renal Inflammation, Renal Nephritis | lupus nephritis | 3.40E-01 | FKBP1A,NR3C1 |
| Cardiac Hypertrophy | hypertrophy of heart cells | 3.50E-01 | CAV1,DACT1,EDN1,IL18,TCF15 |
| Liver Regeneration | regeneration of liver | 3.51E-01 | C3AR1,CAV1,FGF7 |
| Cardiac Hypertrophy | hypertrophy of cardiac muscle | 3.60E-01 | CAV1,DACT1,EDN1,IL18,PDE5A |
| Glomerular Injury, Renal Hyperplasia/Hyperproliferation | hyperplasia of mesangial cells | 3.73E-01 | CDKN1A |
| Renal Inflammation, Renal Nephritis | interstitial nephritis | 3.73E-01 | ACE |
| Liver Proliferation | quantity of hepatocytes | 3.73E-01 | IQGAP2 |
| Cardiac Infarction | acute myocardial infarction | 3.86E-01 | ACE,EDN1,VEGFA |
| Renal Necrosis/Cell Death | apoptosis of kidney cells | 3.86E-01 | CDKN1A,DCN,EDN1 |
| Renal Damage | injury of kidney | 3.86E-01 | BMP7,C3AR1,IL18 |
| Cardiac Hypoplasia | hypoplasia of heart ventricle | 3.86E-01 | NOTCH1,ZFPM2 |
| Renal Necrosis/Cell Death | apoptosis of renal tubular epithelial cells | 3.89E-01 | DCN |
| Liver Damage, Liver Inflammation/Hepatitis | chronic hepatitis B | 3.89E-01 | NR3C1 |
| Congenital Heart Anomaly | situs inversus totalis | 3.89E-01 | LEFTY1 |
| Cardiac Hypertrophy | hypertrophy of cardiomyocytes | 3.92E-01 | CAV1,DACT1,EDN1,IL18 |
| Liver Necrosis/Cell Death | necrosis of liver | 3.96E-01 | CD40,CDKN1A,DUSP4,FKBP1A,IQGAP2,MAP3K8,SLC25A5,SLC26A1 |
| Liver Fibrosis | migration of hepatic stellate cells | 4.05E-01 | EDN1 |
| Liver Cholestasis | cholestasis | 4.20E-01 | CD68,CDKN1A,GJB2 |
| Glomerular Injury, Renal Damage | injury of renal glomerulus | 4.20E-01 | IL18 |
| Liver Failure | failure of liver | 4.22E-01 | EDN1,VEGFA |
| Hepatocellular Carcinoma, Liver Hyperplasia/Hyperproliferation | advanced stage hepatocellular carcinoma | 4.35E-01 | KDR |
| Liver Hyperplasia/Hyperproliferation | proliferation of liver cancer cells | 4.35E-01 | NOTCH1 |
| Hepatocellular Carcinoma, Liver Hyperplasia/Hyperproliferation | development of hepatocellular carcinoma | 4.49E-01 | IQGAP2 |
| Increased Levels of Red Blood Cells | increased quantity of red blood cells | 4.54E-01 | DNASE2,PBX1,STX11 |
| Renal Dysfunction | dysfunction of kidney | 4.64E-01 | ACE |
| Liver Inflammation/Hepatitis, Liver Steatosis | nonalcoholic steatohepatitis | 4.64E-01 | PDE5A |
| Renal Necrosis/Cell Death | cell death of kidney cells | 4.77E-01 | APOBEC3B,BCL6,CDK1,CDKN1A,DCN,DUSP4,EDN1,GRM1,MAP3K8,NLRC4,TP53INP1 |
| Cardiac Dilation | dilation of heart | 4.77E-01 | KLK3 |
| Hepatocellular Carcinoma, Liver Hyperplasia/Hyperproliferation | tumorigenesis of hepatocellular carcinoma | 4.91E-01 | VCAN |
| Cardiac Necrosis/Cell Death | cell viability of cardiomyocytes | 5.17E-01 | EDN1 |
| Liver Damage | hepatotoxicity | 5.17E-01 | IL18 |
| Liver Damage, Liver Inflammation/Hepatitis | hepatitis C | 5.23E-01 | DDX5,FKBP1A |
| Renal Necrosis/Cell Death | apoptosis of kidney cell lines | 5.28E-01 | BCL6,CDK1,DUSP4,GRM1,MAP3K8,NLRC4,TP53INP1 |
| Renal Damage | reperfusion injury of kidney | 5.29E-01 | C3AR1 |
| Liver Damage, Liver Inflammation/Hepatitis | chronic viral hepatitis | 5.38E-01 | DDX5,NR3C1 |
| Congenital Heart Anomaly | failure of heart looping | 5.41E-01 | TBX20 |
| Liver Necrosis/Cell Death | apoptosis of liver cell lines | 5.53E-01 | DUSP4 |
| Liver Hemorrhaging | bleeding of liver | 5.53E-01 | FKBP1A |
| Cardiac Proliferation | proliferation of cardiomyocytes | 5.69E-01 | EDN1,TBX20 |
| Cardiac Dysfunction | dysfunction of heart | 5.83E-01 | DUSP4,NT5E |
| Bradycardia, Cardiac Arrythmia | bradycardia | 5.86E-01 | CHRM2 |
| Cardiac Hypoplasia | hypoplasia of trabeculae carne | 6.17E-01 | NOTCH1 |
| Increased Levels of Creatinine | increased quantity of creatinine | 6.37E-01 | IL18 |
| Liver Necrosis/Cell Death | apoptosis of hepatocytes | 1.00E+00 | CD40,CDKN1A,IQGAP2 |
| Liver Necrosis/Cell Death | cell death of hepatocytes | 1.00E+00 | CD40,CDKN1A,IQGAP2,SLC25A5 |
| Renal Necrosis/Cell Death | cell death of kidney cell lines | 1.00E+00 | APOBEC3B,BCL6,CDK1,DUSP4,GRM1,MAP3K8,NLRC4,TP53INP1 |
| Liver Necrosis/Cell Death | cell death of liver cells | 1.00E+00 | CD40,CDKN1A,DUSP4,IQGAP2,SLC25A5 |
| Liver Damage, Liver Inflammation/Hepatitis | chronic hepatitis C | 1.00E+00 | DDX5 |
| Liver Steatosis | hepatic steatosis | 1.00E+00 | ACE,CAV1,CD40,CDKN1A,CNOT3,IL18,PDE5A |
