# Supplementary material for: Heart function and thoracic aorta gene expression profiling studies of ginseng combined with different herbal medicines in eNOS knockout mice
Source: Sci Rep. 2017 Nov 13;7:15431. doi: 10.1038/s41598-017-15819-2 (PMC5684410; doi:10.1038/s41598-017-15819-2)
Supplement: Supplementary file 2 [file 41598_2017_15819_MOESM2_ESM.doc]

**Supplementary File 2**

**Heart function and thoracic aorta gene expression profiling studies of ginseng combined with different herbal medicines in eNOS knockout mice**

**Yuchen Qian^1^,** ^+^**, Pan Li^1^,**^+^**, Bin Lv^1^, Xiaoqing Jiang^1^, Ting Wang^1^, Han Zhang^1^, Xiaoying Wang^1, 2, *^, Xiumei Gao^1^**

^1^State Key Laboratory of Modern Chinese Medicine, Tianjin University of Traditional Chinese Medicine, Tianjin, 300193, China.

^2^College of Traditional Chinese Medicine, Tianjin University of Traditional Chinese Medicine, Tianjin, 300193, China.

[***Correspondence:**](mailto:*wxy@tjutcm.edu.cn)

[Wang, Xiaoying M.D.](mailto:*wxy@tjutcm.edu.cn)

[Address: 312 Anshanxi Road,Nankai District,Tianjin,P.R.China,300193](mailto:*wxy@tjutcm.edu.cn)

[E-mail: wxy@tjutcm.edu.cn](mailto:*wxy@tjutcm.edu.cn)

**Total RNA isolation**

**1. Tissue Disruption**

1.1 Collect 2–5 mg thoracic aortic rings; wash in cold PBS.

Tissue samples: For good yield of intact RNA, it is very important to obtain tissue quickly and to limit the time between obtaining tissue samples and inactivating RNases.

a. Obtain tissue and remove as much extraneous material as possible, for example remove adipose tissue from heart, and remove gall bladder from liver. The tissue can be perfused with cold PBS if desired to eliminate some of the red blood cells.

b. If necessary, quickly cut the tissue into pieces small enough for either storage or disruption. Weigh the tissue sample.

c. Freeze the sample in liquid nitrogen—tissue pieces must be small enough to freeze in a few seconds. When the liquid nitrogen stops churning, it indicates that the tissue is completely frozen. Once frozen, remove the tissue from the liquid nitrogen and store it in an airtight container below –70°C.

**2.1 Disrupt samples in 600 μL Lysis/Binding**

a. Place 10 volumes of Lysis/Binding Buffer per tissue mass into aplastic weigh boat or tube on ice.

b. Using a prechilled metal spatula, scrape the powdered tissue into theLysis/Binding Buffer, and mix rapidly.

c. Transfer the mixture to a vessel for homogenization and process themixture to homogeneity.

**2. Organic Extraction**

**2.1 Add 1/10 volume of miRNA Homogenate Additive, incubate 10 min on ice**

a. Add 1/10 volume of miRNA Homogenate Additive to thetissue lysate (or homogenate), and mix well by vortexing or invertingthe tube several times. For example, if the lysate volume is 300 μL,add 30 μL miRNA Homogenate Additive.

b. Leave the mixture on ice for 10 min.

**2.2 Extract with a volume of Acid-Phenol: Chloroform equal to the initial lysate volume**

a. Add a volume of Acid-Phenol:Chloroform that is equal to the lysatevolume before addition of the miRNA Homogenate Additive.

b. Vortex for 30–60 sec to mix.

c. Centrifuge for 5 min at maximum speed (10,000 x g) at roomtemperature to separate the aqueous and organic phases. Aftercentrifugation, the interphase should be compact; if it is not, repeatthe centrifugation.

**2.3 Recover the aqueous phase; transfer the aqueous phase to a fresh tube**

Carefully remove the aqueous (upper) phase without disturbing thelower phase, and transfer it to a fresh tube. Note the volume removed.

**3. Final RNA Isolation**

**3.1 Add 1.25 volumes 100% ethanol, and mix thoroughly**

Add 1.25 volumes of room temperature 100% ethanol to the aqueousphase

**3.2 Pass the lysate/ethanol mixture through a Filter Cartridge**

a. For each sample, place a Filter Cartridge into one of the CollectionTubes supplied.

b. Pipet the lysate/ethanol mixture (from the previous step) onto theFilter Cartridge. Up to 700 μL can be applied to a Filter Cartridge ata time, for samples larger than this, apply the mixture in successiveapplications to the same filter.

c. Centrifuge for ~15 sec to pass the mixture through the filter.Centrifuge at RCF 10,000x g (typically 10,000 rpm). Spinningharder than this may damage the filters.Alternatively, vacuum pressure may be used to pass samples throughthe filter.

d. Discard the flow-through, and repeat until all of the lysate/ethanolmixture is through th e filter. Reuse the Co llection Tube for thewashing steps.

**3.3 Wash the filter with 700 μL miRNA Wash Solution 1**

Apply 700 μL miRNA Wash Solution1 (working solution mixed withethanol) to the Filter Cartridge and centrifuge for ~5–10 sec or use avacuum to pull the solution through the filter. Discard the flow-throughfrom the Collection Tube, and replace the Filter Cartridge into thesame Collection Tube.

**3.4 Wash the filter twice with 500 μL Wash Solution 2/3**

a. Apply 500 μL Wash Solution 2/3 (working solution mixed withethanol) and draw it through the Filter Cartridge as in the previousstep.

b. Repeat with a second 500 μL aliquot of Wash Solution 2/3.

c. After discarding the flow-through from the last wash, replace theFilter Cartridge in the same Collection Tube and spin the assemblyfor 1 min to remove residual fluid from the filter.

**3.5 Elute RNA with 100 μL 95°C Elution Solution or Nuclease-free Water**

Transfer the Filter Cartridge into a fresh Collection Tube (providedwith the kit). Apply 100 μL of pre-heated (95°C) Elution Solution ornuclease-free water to the center of the filter, and close the cap. Spin for~20–30 sec at maximum speed to recover the RNA.Collect the eluate (which contains the RNA) and store it at –70° C.

**The results of RNA quality control**

| **Sample ID** | **Concentration (µg/µl)** | **A260/**  **A280** | **Volume**  **(µl)** | **Total Mass（µg）** | **28S/18S** | **RNA Integrity Number** | **QCResult** |
| --- | --- | --- | --- | --- | --- | --- | --- |
| C57-1 | 0.0795 | 2.17 | 95 | 8 | 1.5 | 9.0 | Pass |
| C57-2 | 0.0545 | 2.14 | 95 | 5 | 1.6 | 9.3 | Pass |
| C57-3 | 0.1243 | 2.14 | 95 | 12 | 1.6 | 9.2 | Pass |
| eNOS KO-1 | 0.0410 | 2.13 | 95 | 4 | 0.8 | 7.0 | Pass |
| eNOS KO-2 | 0.0447 | 2.14 | 95 | 4 | 1.1 | 8.4 | Pass |
| eNOS KO-3 | 0.0885 | 2.17 | 95 | 8 | 1.1 | 8.2 | Pass |
| SFI-1 | 0.1241 | 2.03 | 100 | 12 | 1.4 | 8.6 | Pass |
| SFI-2 | 0.1855 | 2.13 | 100 | 19 | 1.0 | 8.4 | Pass |
| SFI-3 | 0.3584 | 2.15 | 100 | 36 | 1.2 | 8.1 | Pass |
| SMI-1 | 0.1437 | 2.12 | 100 | 14 | 1.0 | 8.5 | Pass |
| SMI-2 | 0.2403 | 2.13 | 100 | 24 | 0.8 | 7.2 | Pass |
| SMI-3 | 0.3846 | 2.16 | 100 | 38 | 1.1 | 8.3 | Pass |
| VAL-1 | 0.1056 | 2.17 | 95 | 10 | 0.8 | 7.2 | Pass |
| VAL-2 | 0.0435 | 2.08 | 100 | 4 | 1.2 | 7.8 | Pass |
| VAL-3 | 0.0920 | 2.11 | 100 | 9 | 1.0 | 7.6 | Pass |
